# Why and how the early-life environment affects development of coping behaviours

**DOI:** 10.1007/s00265-018-2452-3

**Published:** 2018-02-09

**Authors:** M. Rohaa Langenhof, Jan Komdeur

**Affiliations:** Behavioural Physiology and Ecology Group, Groningen Institute for Evolutionary Life Sciences, University of Groningen, Groningen, Netherlands

**Keywords:** Early-life environment, Developmental processes, Personality, Coping, Intergenerational effects, Parental effects

## Abstract

Understanding the ways in which individuals cope with threats, respond to challenges, make use of opportunities and mediate the harmful effects of their surroundings is important for predicting their ability to function in a rapidly changing world. Perhaps one of the most essential drivers of coping behaviour of adults is the environment experienced during their early-life development. Although the study of coping, defined as behaviours displayed in response to environmental challenges, has a long and rich research history in biology, recent literature has repeatedly pointed out that the processes through which coping behaviours develop in individuals are still largely unknown. In this review, we make a move towards integrating ultimate and proximate lines of coping behaviour research. After broadly defining coping behaviours (1), we review why, from an evolutionary perspective, the development of coping has become tightly linked to the early-life environment (2), which relevant developmental processes are most important in creating coping behaviours adjusted to the early-life environment (3), which influences have been shown to impact those developmental processes (4) and what the adaptive significance of intergenerational transmission of coping behaviours is, in the context of behavioural adaptations to a fast changing world (5). Important concepts such as effects of parents, habitat, nutrition, social group and stress are discussed using examples from empirical studies on mammals, fish, birds and other animals. In the discussion, we address important problems that arise when studying the development of coping behaviours and suggest solutions.

**Electronic supplementary material:**

The online version of this article (10.1007/s00265-018-2452-3) contains supplementary material, which is available to authorized users.

The behaviours that animals display to respond to challenges in their environment—whether to avoid a threat or to utilise an opportunity—relate directly to their ability to survive and reproduce. This is especially the case in a quickly changing world (Lapiedra et al. 2017). As such, understanding these behaviours has been a topic of study not only in behavioural biology, but also in evolutionary and conservation biology. Coping is commonly considered as the behavioural and physiological efforts to master a challenging situation (Koolhaas et al. 1999). Despite a long research history, very little is known about the way coping behaviours develop in individuals (Belsky and Pluess 2009a; Rao et al. 2010; Stamps and Groothuis 2010; Gracceva et al. 2011; Groothuis and Trillmich 2011; Cowan et al. 2016). Much attention is currently focussed on finding evidence of individual differences in coping behaviours across different species (Bell and Stamps 2004; Dall et al. 2012; Ogden 2012), understanding the active-reactive axis on which (some of) such coping behaviours seem to fall (Sloan Wilson et al. 1994; Koski 2011; Pascual and Senar 2014), explaining the evolutionary mechanisms underlying individual differences (Dingemanse et al. 2002; Adriaenssens and Johnsson 2011; St-Hilaire et al. 2017) and integrating its implications for ecological and behavioural studies. While it is very important to correlate developmental influences with one or more behavioural traits and discover variables that shape adult coping behaviour, such lines of research do not provide sufficient clarity on the proximate and ultimate aspects of the development of coping behaviours (Groothuis and Trillmich 2011).

A great deal of developmental research has been devoted to understanding whether and how experiences in ontogeny shape behavioural development later in life, yet insufficient attention has been paid to why and how such cross-time influences should characterise animal (Skinner and Zimmer-Gembeck 2007; Groothuis and Trillmich 2011; Trillmich and Hudson 2011) or even human (Belsky 2007; Ellis and Boyce 2008; Haun et al. 2013) development, or how natural selection structures the early-life effects on development (Ellis and Boyce 2008). Despite extensive study on specific stressors and behaviours, no overarching developmental framework currently exists to explain why or how animals develop the strategies with which they respond to their environment (Skinner and Zimmer-Gembeck 2007; Hengartner 2017).

Within the development of coping behaviours (defined for the purpose of this review as the behaviours that individuals exhibit aimed at responding to environmental challenges), there is an important unexplored niche in the ways through which the environment during early-life development shapes coping behaviours used later in life (Trillmich and Hudson 2011; Miranda 2017; Zidar et al. 2017). Yet environmental stimuli during this early-life period are extremely relevant, as costs, limitations, opportunities and a variety of external factors experienced during ontogeny affect developmental processes that lead to coping behaviours (Skinner and Zimmer-Gembeck 2007; Stamps and Groothuis 2010). Environmental influences may begin prenatally and may be amplified postnatally as individuals come to occupy different niches within their surroundings, interact with conspecifics and cope with environmental challenges (Hudson et al. 2011; Trillmich and Hudson 2011). The pathway to either vulnerability or resilience is influenced by a complex matrix (Cicchetti 2010), in which environmental factors such as the social context, past and current experiences and timing of the experiences are key factors (Fawcett and Frankenhuis 2015). As such, the early-life environment has complex and long-lasting effects on later life behaviour (Burton and Metcalfe 2014; Cowan et al. 2016; Carlson 2017), directly affecting both the type and the dynamic range of behaviours individuals have available later in life (Rödel and Monclús 2011).

Especially in the light of global change and the increasing need of animals (and humans) to adapt to ever changing environments, it is essential to study coping behaviour and its causal factors, as coping behaviour directly relates to animals’ ability to adapt to novel and changing environments (Taborsky 2017). While of course genetics is important for our understanding of the building blocks underlying coping behaviour, in the field of coping and animal personality (see “Coping behaviours: definitions and precursors” section), much of this work has already been done (Bouchard and Loehlin 2001; van Oers et al. 2005), while the complexity of developmental processes has not received as much attention as it should. One might argue that in times of great change, behaviour is the most immediate and effective way for individuals and populations to respond to environmental challenge (Kappeler et al. 2013), and the processes that lead to resilience and flexibility in coping behaviours in individuals become essential to our understanding of larger level trends in response to environmental pressure. Understanding the interplay between environmental influences and developmental processes assists in predicting which environmental influences can be harmful (and under which conditions), which types of maladaptive coping behaviours may be reversed and how interventions can facilitate such reversibility.

In this review, we offer an environmental perspective on the development of coping and simultaneously consider the process from an evolutionary angle—a novel synthetic approach that has been lacking so far. Without going too deeply into neurobiological details, we highlight the importance of the early-life environment on the development of coping behaviours and extensively review relevant literature to further an understanding of both ultimate (why) and proximate (how) causes of this essential role of the early-life environment in the way animals respond to challenges in their surroundings. The early-life environment is defined here as the non-genetic biotic or abiotic external factors that affect an animal in the period from conception to the time it can survive independently.

The review includes (1) a working definition of coping behaviours and a brief overview of components that are precursors to successful coping, (2) evolutionary reasons for the development of coping to be strongly affected by the early-life environment, (3) developmental processes through which the early-life environment affects later-life coping behaviours, (4) environmental influences shown to affect these developmental processes and (5) non-genetic intergenerational transmission of coping behaviours, an overarching topic that concerns both why and how coping behaviours are affected by the early-life environment, which we discuss in the context of behavioural adaptation to a rapidly changing world (see Fig. 1). In order to provide an extensive review of the literature, the ISI database and Google Scholar were searched, and all studies that matched inclusion criteria were listed in a comprehensive overview (see Suppl. material). A detailed overview of the reviewed literature can be found in the Supplementary material. This approach provides the starting parameters for a model to understand more closely why some environmental factors affect development of some coping behaviours differently than others, during different developmental stage and in what direction. With this review, we attempt to contribute to a better understanding of the challenges individuals face in adapting to new environments. To the best of our knowledge, this is the first review to attempt combining early-life influences with the processes through which coping behaviours are established.

**Fig. 1:**
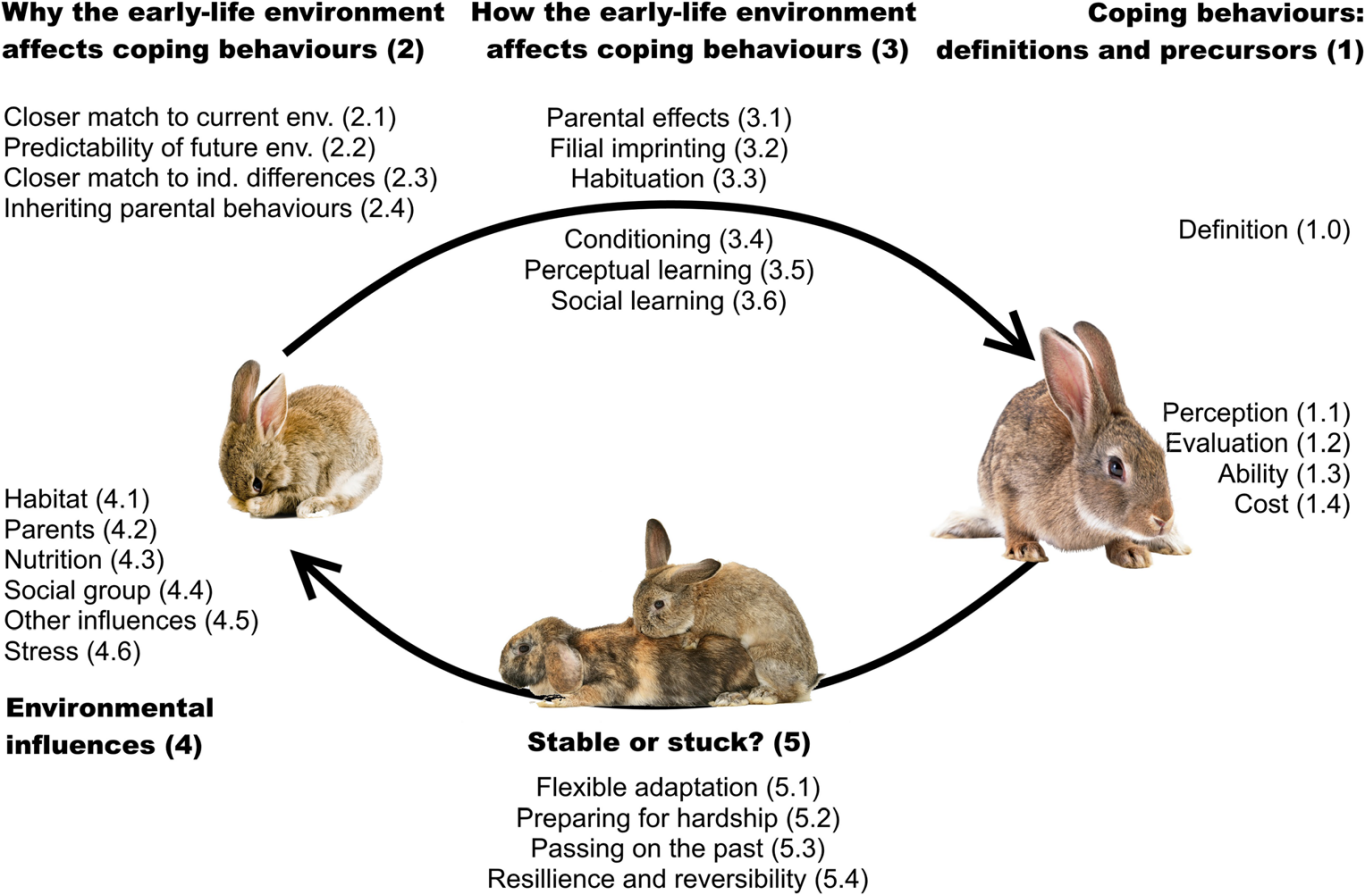
Schematic overview detailing ultimate and proximate causes of sensitivity of developmental processes to the early-life environment, mapped around the life cycle of a group-living mammal, the European rabbit (*Oryctolagus cuniculus*). Numbers between brackets correspond to paragraph sections in the main text. Images from open source stock

## Coping behaviours: definitions and precursors

In order to survive and meet their basic needs, all animals constantly interact with their environment. They search to acquire food and other resources, watch out for predators and other dangers, and secure a safe place to rest. They interact with animals of their own species, attempt to find a suitable mate and take care of their offspring. Although coping, animal personality, temperament and behavioural syndromes differ importantly in underlying theory and context (Stamps and Groothuis 2010), all are commonly used to study how animals react to challenges in their surroundings. Coping is commonly considered as the behavioural and physiological efforts to master a challenging situation (Koolhaas et al. 1999), while behavioural syndrome is often defined as individual differences in behaviour patterns that are either correlated across time or contexts (Sih and Bell 2008; Dochtermann and Dingemanse 2013), and animal personality, similar to coping styles (Réale et al. 2007), is commonly defined as underlying behavioural tendencies that differ across individuals, that are consistent within individuals over time and that affect the behaviour that is expressed in different contexts (Caspi et al. 2005; Réale et al. 2007; synthesised in Stamps and Groothuis 2010). While coping and personality have been linked many times (McCrae and Costa 1986; Jang et al. 2007; Carver and Connor-Smith 2010; Kaiseler et al. 2012) and are sometimes used interchangeably (Melotti et al. 2011), they cannot be considered identical, as personality makes assumptions on cross-context and cross-time repeatability (Dingemanse et al. 2012), whereas coping does not.

Due to the recent popularity of studies of coping and animal personality, terminology used for the behaviours animals use to respond to their environment can be confusing, overlapping or inconsistent (Carter et al. 2013). As a result, the same behaviour is often studied from many different perspectives, sometimes with slightly different connotation, and it is often unclear what the exact distinction is between a behaviour and a behavioural syndrome, coping style or personality, especially in species or populations where behaviours are tightly correlated into behavioural suites. Exploration is an excellent example: some studies consider exploration to be one aspect of animal personality (Wolf et al. 2007; Minderman et al. 2009; Schuett et al. 2013), or part of a behavioural syndrome (Bell and Sih 2007; Dingemanse et al. 2007; Wisenden et al. 2011); others group it in with the active-passive axis of coping (Janczak et al. 2003), yet others study it as a single behaviour (Dingemanse et al. 2002; Mettke-Hofmann et al. 2002). Depending on the aim of the study, all of these approaches can be correct. However, distinguishing whether a behaviour is part of a behavioural suite or a coping axis or a personality suite or only partially or not at all falls far outside the scope of this review, especially since the answers to such questions differ widely between species and even populations within species (Carter et al. 2013).

For the purpose of this review, we use the more generic, inclusive term “coping behaviours”, defined here as “the behaviours that individuals exhibit aimed at responding to environmental challenges”, including but not limited to exploration, avoidance, approach, boldness, shyness, aggression and response to novelty. Where they are clearly relevant to coping behaviours, we also discuss animal personality traits such as anxiety, stress responsiveness or impulsivity. We furthermore include expressions of sociality, as social behaviour is an important component to coping with challenges for group-living animals (Fischer et al. 2015). For the sake of a comprehensive and balanced review, studies were evaluated on a case-by-case basis and only included if the behaviour could reasonably be considered a behavioural response to an environmental challenge. As such, we often (but not always) included studies on exploration as well as those on personality and excluded studies on genetics, neurochemistry and theoretical models. This allows us to consider all relevant empirical work dealing with behaviours currently thought to be involved in coping with environmental challenges, regardless of the terminology or framework used in the study, while still excluding generic behaviour that is not in any obvious way related to immediate response to environmental challenge.

It should be noted that in order to create the broad scale experimental design necessary to study animal personality, coping styles or behavioural syndromes, testing is often done under laboratory conditions with stock animals that, while creating genetic and environmental homogeneity between studies, may be unable to exhibit behavioural responses that are ecologically or evolutionary relevant (Koolhaas et al. 2011; Carter et al. 2013; Junco 2017). Laboratory conditions, whether experienced by the parents, in early-life, or during the experiment, warp the behaviours with which animals respond. However, only very little developmental work has been done under natural conditions (Rödel et al. 2017), and as long as the aforementioned caveat is kept in mind, non-natural experimental conditions still provide important insights into the underlying developmental mechanisms of coping.

When coping behaviours are considered as the decision an animal makes with regards to the behaviour it will use to mediate a challenge presented by its environment, as follows from our definition, it becomes relevant to consider precursors to that decision. For all animals, it can be hypothesised that successful adaptation to environmental conditions depends on (at least) four pillars: *perceiving* a need for a response (1), *evaluating* an effective response (2), *ability* to give that response (3) and *paying the cost* for that response (4). As a thought experiment: in order to successfully cope with a larger animal encroaching into its habitat, a potential prey has to have a sensory awareness of the larger animal, followed by the perception (1) that this animal is either harmless or dangerous. If the latter is the case, the prey has to evaluate (2) whether hiding or running away is the most effective way to respond in this situation and to this type of predator, and (3) whether it is physically capable of running fast enough to get away. If it does hide or run, it can lose foraging time and valuable resources or encounter other dangers. These state- and condition-dependent costs need to be factored into the decision in favour of a particular coping behaviour.

There is strong selection on traits making up each of these four pillars, as each directly factors into an individual’s coping behaviour, and there are immediate and possibly life-threatening consequences to responding with ineffective behaviours (Stirling et al. 2002; Adriaenssens and Johnsson 2013). As such, these precursors to coping behaviour, like coping behaviours themselves, are strongly influenced by early-life environment developmental process. Below, we briefly detail these four precursors and their evolutionary relationship to the early-life developmental period, in order to more fully understand the relationship between coping behaviours and the early-life environment.

### Perception

Perceiving a threat is a first and necessary step to coping (Edenbrow and Croft 2013), whether that perception happens on a conscious or unconscious level (Lovibond and Shanks 2002). Animals cannot respond to dangerous situations that their sensory systems cannot perceive (Shettleworth 2001; Guesdon et al. 2011). Herein also lays a vulnerability, one that has been widely explored within the domain of psychology (Brewer et al. 2007; Volk et al. 2010; Arran et al. 2014) but surprisingly much less considered in animal biology. The perception of a threat can be inaccurate, thereby preventing successful coping from the start. Individuals may fail in sensory perception of a threat or fail to perceive a situation accurately enough to consider it a threat. For example, iguanas who were confronted with an approaching human, moved earlier, ran earlier and ran farther when the human’s face was exposed versus covered by hair, as a covered face gave the conflicting stimulus of both approaching and retreating (Burger and Gochfeld 1993). Alternatively, individuals may perceive a threat where there is none, for example in animals that are over-easily startled in response to novel but non-threatening sounds or visuals such as digital ringtones or billboards (King et al. 2003; Potvin 2017), or to human outdoor recreation (Tablado and Jenni 2017), which can lead to (social) stress and the many negative health consequences that come from long-term stress (Moberg 1985; Tapp and Natelson 1988; Blanchard et al. 2001; Cockrem 2007).

In order to perceive threats more accurately, animals use several strategies, such as becoming more sensitive to predator behaviour and specific morphological traits to distinguish one type of predator from another (Stankowich and Blumstein 2005). Another effective strategy is to increase vigilance. Perceiving the environment with accuracy for an extended period of time in order to detect potential dangers is costly, as it takes away from other activities such as foraging, which some species of animals can minimise by increasing group size and sharing vigilance (Eilam et al. 2011). As a result, many group-living animal species have evolved within-group sharing of acquired and evaluated information (Liddell et al. 2004; Magrath et al. 2015; Wingfield 2015) and a sensitivity to alarm displays from conspecifics (Liddell et al. 2004; Brown et al. 2006; Rieucau et al. 2014) as well as cues directly from predators (Rieucau et al. 2014). Detection of such cues does not need to be conscious (Liddell et al. 2004).

For young animals, there is little room for trial-and-error in distinguishing between friend and foe, as they are often vulnerable to predation from predator species as well as their own kind. For this reason, there is strong selection for young animals to acquire effective risk appraisal (or risk assessment) early in life. As a result, the situations they perceive as a threat or a danger later in life is closely linked to early-life conditions. An adult responding aggressively in certain situations may be doing so because their early-life environment has induced a heightened threat perception, or may respond impulsively because their experiences did not prepare them for the possibility of a predator (Bell et al. 2011; El Balaa and Blouin-Demers 2011). Although it is widely recognised in animal biology that threat perception is an important aspect of animal functioning (Burger and Gochfeld 1993; Kirschvink 2000; Chamaillé-Jammes et al. 2014; Rieucau et al. 2014), threat perception and differences therein based on early-life environment are rarely linked to the development of coping behaviours (Brown et al. 2013; Zidar et al. 2017). In part, this is because risk perception (degree of “fear”) is difficult to study in animals (Stankowich and Blumstein 2005) and especially difficult to separate from the subsequent response.

### Evaluation

Evaluating an effective response to a situation can be defined simply as carrying out any response that successfully mediates the challenging situation to where there is no longer a threat or opportunity. When an individual does not correctly estimate which response should be given, it creates a mismatch between behaviour and environment that can be fatal to the animal. This was observed, for example, when captive-bred angelfish (*Pterophyllum scalare*), who had never experienced a predator before, were challenged to respond to predator cues, to which they responded less appropriately than wild-bred fish with past experience of a predator (El Balaa and Blouin-Demers 2011). Such a mismatch also occurs when individuals respond too readily to possible threats. Responding only to individuals of a predator species that display sufficiently threatening behaviour allows prey species to minimise energy expenditure and other costs of predator avoidance, which is especially relevant if the predator is common but attacks are infrequent (Papworth et al. 2013).

It must be noted that the coping responses that animals display are often non-conscious and part of a stimulus-response bond (Liddell et al. 2004), a pathway that has become engrained through a myriad of developmental processes (see “How the early-life environment affects the development of coping/developmental processes affecting coping behaviours” section). Yet there is indication that animals choose from multiple available strategies, as outlined below, when environmental conditions incite them to respond to a threat (Benus et al. 1991; Belsky 2007; Mathot et al. 2012). Which strategy is estimated as the most effective is dependent upon current environmental conditions as well as past experience and the animal’s personal success rate with the available coping strategies. The threat-sensitive predator avoidance model (Bishop and Brown 1992; Brown et al. 2006; Chamaillé-Jammes et al. 2014; Rieucau et al. 2014) predicts that animals should take into account perceived predation risk to balance the intensity of their antipredator response. Research on cichlids showed that when threatened, isolated individuals exhibited reduced time moving and foraging than individuals in shoals, and small shoals exhibited a higher response threshold than large shoals (Brown et al. 2006). Similarly, wild-caught herrings provided the strongest avoidance reactions when exposed to versatile predator sensory cues (Rieucau et al. 2014). These findings and others (Bishop and Brown 1992) indicate that response patterns are flexible and situation dependent, and subject to natural selection processes.

As much as threat perception, threat evaluation is dependent at least to a degree on experiences during early life (Olff et al. 2005). Familiarity with the situation and habituation, both of which play a role in estimating effective coping behaviours, are built through either personal experience with similar situations earlier in life (Snell-Rood et al. 2013), or learning from others who previously experienced the situation (Brown et al. 2006; Rieucau et al. 2014). Domestically raised animals, for example, no longer perceive humans as threats, whereas many wild animals do. While they perceive similar cues, they evaluate the presence of a human differently and so display different coping behaviours. The ability to evaluate which response to take under different circumstances is especially relevant in coping with novelty, when little previous experience is available (Tang et al. 2011).

### Ability

Even when a challenge has been perceived and an appropriate response has been evaluated, successful coping is not guaranteed. Animals may not be able to give the response that is most effective, due to physical or developmental constraints or negative experiences earlier in life (Long 1990; Leichty et al. 2012). Stressors or deprivation of nutrients, care, personal experience or parental example early in life may have made it impossible to give an adaptive response. For example, the nest environment in young rats was shown to affect ontogeny of personality types, with heavier individuals being more bold and more explorative than lighter individuals, and individuals from both small and large litters being more anxious than individuals from medium-sized litters (Rödel and Meyer 2011). Thus, the appropriate coping behaviour for a rural rat facing food shortage might be to go into an urban area to forage, but because the rat in question had a low birth weight and came from a large litter, its coping behaviours were developed towards the shy and anxious range, leaving it behaviourally unable to cope with the noise and novelty of an urban environment. A rat with even slightly different early-life circumstances, by contrast, might make the transition to the new environment and survive the food shortage. In addition to stressors or deprivation, which focuses specifically on negative early-life events, development of behavioural processes may of necessity have canalised (Hermanussen et al. 2001; Dochtermann and Dingemanse 2013) in a particular direction that excludes the desired behaviour.

### Cost

Finally, if an animal responds with a particular coping behaviour, it must be able to incur the costs of that behaviour; otherwise, the coping response will lead to a loss of fitness rather than a gain, either immediately or over a longer time span. Such costs can come in many forms. An animal that responds to the appearance of a competitor at the feeding ground with flight, freezing or hiding loses the opportunity to forage and obtain resources (McArthur et al. 2014). An animal that responds with aggression, on the other hand, may incur cost to its physical health sustained in fighting displays (Marler and Moore 1988; Johnstone 2001; Lane and Briffa 2017). If the aggression is part of a behavioural construct, where the same coping behaviour is habitually displayed across contexts (Bell and Stamps 2004), the individual may incur costs when the same aggression effectively displayed towards a rival male becomes lethal when displayed towards a predator, or alternatively, when effective aggressive behaviour towards predators may incur costs on other coping behaviours such as vigilance (Hess et al. 2016).

Life-history aspects such as offspring weight (Ferrari et al. 2015), parental care (Budaev et al. 1999; Reddon 2012; van Oers et al. 2015), rank within the social structure (Verbeek et al. 1999) and predation pressure are important in determining physical strength as well as resultant behavioural flexibility across an individual lifetime, and so affect whether or not an animal can afford the cost of a particular coping behaviour or behavioural syndrome (Fish et al. 2004; Rödel and Monclús 2011). For example, physically strong individuals, as well as animals reared in nutritionally challenging environments, may have higher chances of survival if they develop coping behaviours involving boldness, exploration and high levels of activity (Krause et al. 2009; Noguera et al. 2015). However, for physically weak animals, or those living under high predation pressures, active and bold coping behaviours may be extremely costly, illustrated by the inducement of a boldness-aggression behavioural correlation in sticklebacks under predation pressure (Bell and Sih 2007), which suggests that in this high-risk environment, it is adaptive for animals to be bold only if they are also (able to be) aggressive. In addition, the cost of a coping behaviour relates to the experience an animal has performing this behaviour, as it is considered risky to attempt novel coping behaviours when faced with a threat (King et al. 2003; Martin and Réale 2008; Rothwell et al. 2011).

## Why the early-life environment affects the development of coping

From an evolutionary perspective, coping behaviour is an animal’s first line of defence against challenging circumstances—whether it be to avoid a threat or take advantage of a rare resource. Failure to cope is likely to cause negative consequences for the individual (Koolhaas et al. 1999), such as reduced health (Olff et al. 1993; Taylor 2010) and immuno-competence (O’Mahony et al. 2009). As such, natural selection processes are expected to target development of those coping behaviours that increase the individual’s ability to accurately respond to threats and to most effectively utilise opportunities to its benefit. Indeed, there is ample evidence for heritability in coping behaviours, although values tend to differ per species, behaviour and type of measurement (Benus et al. 1991; Dingemanse et al. 2002; Jang et al. 2006; Rice 2008).

Recently, now that researchers start to work increasingly from a postgenomics outlook, the environment is more and more considered to be at least as crucial as the DNA sequence for constructing the (behavioural) phenotype, and as a source of information in predicting the phenotype (Schoener 2011; LaFreniere and MacDonald 2013). It has become clear that environmental factors, experienced even during the very earliest stages of life, have the potential to cause irreversible developmental changes (Burton and Metcalfe 2014; Cowan et al. 2016; Carlson 2017), allowing individuals to acquire a variety of phenotypes with long-term consequences for performance (Gilbert 2001). In this section, we review why, from an evolutionary point of view, the early-life environment in particular is so essential for the development of coping behaviours.

A key concept in this context is phenotypic plasticity, sometimes defined as the flexibility of developmental processes to generate a variety of different phenotypes from the same genotype, through sensitivity to early-life environmental input (Debat and David 2001; Dingemanse et al. 2010; Mery and Burns 2010; Nettle and Bateson 2015). Its opposite, canalisation, concerns developmental processes that produce the same phenotype regardless of variability of environmental input or genotype (Waddington 1942; Debat and David 2001; Siegal and Bergman 2002). Within animal personality research, these terms are sometimes also used to indicate an animal’s ability to display a variety of responses dependent on the situation, or alternatively to have behaviours linked to where they are likely to display the same behaviour across time and context. The extent to which a (behavioural) trait is plastic or canalised depends on the species, although canalisation is often found more strongly in behaviours that are essential to survival, where there is very little margin for error (Debat and David 2001).

### Closer match to current environment

The most straightforward reason why the development of coping behaviours is strongly affected by early-life conditions is to allow for a closer match between coping behaviour and juvenile environment than if behavioural development were solely determined by genes (Grether 2005; Lof et al. 2012; Taborsky 2017). Natural environments are fluid and can fluctuate strongly with respect to climate, available resources, habitat size and connectivity, predation and competition pressures, social dynamics and more. When young animals are born, they have little conscious knowledge of the surroundings that will define their survival, the challenges ahead, or exactly what strategies will be effective in dealing with the predators, the social hierarchy and the resources they will find in their habitat. From an evolutionary perspective, there is therefore a distinct benefit to maintaining developmental processes that facilitate fast and targeted learning in the very first stages of life, as well as processes that fine-tune coping behaviours to existing environmental conditions. As the early-life environment can be almost indistinguishable from life history, an additional advantage is a closer match to life-history aspects such as birth weight and number of offspring (Wolf et al. 2007; Edenbrow and Croft 2011; Niemelä et al. 2012; Hengartner 2017). Developmental processes that are sensitive to input from the immediate surroundings allow organisms a degree of flexibility and adaptability across generations, and increase the chances that young are capable of responding quickly and appropriately to surroundings that they do not yet have the personal experience with. Young animals with effective and efficient coping behaviours are more likely to survive to adulthood and to do so with a higher body mass than those with less well-adjusted behaviours, which has been shown to impact later life success (Lindström 1999; Noguera et al. 2015).

Heightened sensitivity of development to conditions experienced during early life may increase an animal’s chances of developing behaviours that are functional within their surroundings. If young individuals experience an unsafe environment in which their life and health are in constant danger, it is beneficial to them to develop keener senses (Aron et al. 2012), build a cognitive database on hiding places and learn to respond to unexpected stimuli by freezing in place or bolting for cover. However, if they experience a relatively safe environment, there is a greater benefit to engaging a new situation and exploring unexpected stimuli, as there is little risk and a better opportunity of finding additional resources. In line with this reasoning, a large body of evidence indicates that the environment young animals experience is essential in determining how they develop their coping behaviours (see “What in the early-life environment affects later-life coping behaviours” section). For example, rodent young who grow up in a large family context develop a greater awareness of social subtleties than young who receive only a little social stimulation (Ahern and Young 2009; Branchi 2009), and cichlids (*Neolamprologus pulcher*) raised in larger social context developed greater social competence (Fischer et al. 2015). Similarly, cichlids raised in the presence of predator fish compared to environments without predators develop more sensitive behaviour (Fischer et al. 2017). An excellent example of adaptiveness of early-life conditions is found in a study on zebra finches (*Taeniopygia guttata*). In this species, juveniles generally learn foraging skills from their parents. When juveniles were exposed to developmental stress, however, they switched to learning foraging skills exclusively from biologically unrelated adults. Stress has been suggested as an environmental cue (Farine et al. 2015) and may represent an honest signal that parental coping behaviours are insufficient and should not be copied.

However, too much sensitivity to details of the early-life environment can in turn lead to maladaptation, as environments are naturally changeable and prone to stochasticity. It is essential that developmental processes are maximally sensitive to those cues that are predictive of the environment (for example, low winter temperatures caused by a mini-ice age), and at the same time minimally sensitive to those that are caused by short-term stochastic processes (an especially chilly week). One way to navigate this trade-off is through sensitivity to maternal stress, which is increasingly argued as potentially beneficial to offspring (Sheriff et al. 2017). Indiscriminate sensitivity causes vulnerability to behavioural mismatch with the environment later in life (Raubenheimer et al. 2012; Jensen et al. 2014). Being able to differentiate between predictive and stochastic early-life input is a challenge to developmental processes, especially due to the short developmental window available to most species (weeks to months), and especially in times of great change (Roberts et al. 2011) where it cannot be assumed that parental environment equates offspring environment. Evolutionary speaking, it is therefore essential that developmental processes maintain a balance between robustness and adaptiveness (see also “Resilience and reversibility” section). There is a need for sufficient sensitivity and specificity to relevant environmental cues, while at the same time minimising judgement errors, which sensitivity to early-life environments can in some circumstances provide (see “Stable or stuck across generations?” section).

### Predictability of later-life environment

Under certain circumstances, the early-life environment can reliably predict the later-life environment (McLinn and Stephens 2006; Branchi and Cirulli 2014), much like a “weather forecast” of the conditions in which an animal will mature, making it adaptive for an animal to develop a phenotype suitable for this expected environment (external predictive adaptive responses) (Nettle et al. 2013). Practically, this may translate to physical changes in body size or developmental time (Beckerman et al. 2007; Niemelä et al. 2012), but also to behavioural changes in foraging strategies, aggression, shyness, social behaviour, activity and exploration (Wells 2007b). Some theoretical models suggest that it is only possible for species to evolve developmental sensitivity to early-life cues when developing individuals get accurate cues about their future adult environment (Proulx and Teotónio 2017). Some empirical work, however, seems to point towards a more complex mechanism that takes into account multiple life-history aspects (Biro and Stamps 2008; Burton and Metcalfe 2014). Gaining information about future adult environment is considered more important in some species than in others, depending on the life-history aspects relevant to species, although there is still discussion about the relevance of external prediction especially in longer lived species (Burton and Metcalfe 2014).

One way for offspring to predict conditions of their later-life environments is through parental cues. Parents can adjust the phenotype of their offspring to match the local environment through anticipatory parental effects (APEs), so as to increase the fitness of both parents and offspring. When wild cavy mothers (*Cavia porcellus*) experienced an unstable social environment, her male offspring developed a behavioural camouflage strategy, hypothesised to be beneficial at the time of social challenge (Siegeler et al. 2017). In Japanese quail (*Coturnix japonica*), pre-natal stress experienced by the mother resulted in inheritance of the same stress-coping traits in offspring across neuroendocrine, physiological and behavioural traits. These responses have been suggested as adaptations to preparing offspring for a future environment in which the same stressors are experienced (Zimmer et al. 2017).

The effect of parental cues is predicated on the idea that parental environment is a reliable predictor of offspring environment, which is not always the case (Burgess and Marshall 2014). Parental effects on offspring coping behaviours do not always favour the offspring, but in some cases seem to exclusively benefit the mother (Wells 2007a; Sheriff and Love 2013). In some cases, the early-life environment itself is shaped by the parents through parental provisioning, nest building and other environment-changing behaviours, which increases the predictive validity of parental signalling.

Within parental cues about the environment, especially parental predator warnings are important in demonstrating the evolutionary relevance of sensitivity to the early-life environment. Offspring of female fall field crickets (*Gryllus pennsylvanicus*) exposed to a predator spider during gestation showed greater anti-predator immobility in response to spider cues than offspring of non-exposed females. “Warned” offspring then survived better when faced with spiders (Storm and Lima 2010), clearly demonstrating the adaptive value of this parental effect. Similarly, in common lizards (*Zootoca vivipara*), maternal exposure to snake cues during gestation affected juvenile behaviour and dispersal towards increased risk avoidance strategies (Bestion et al. 2014). Studies such as these show that cues from mothers during gestation can trigger adaptive anti-predator responses aimed at increasing offspring survival, although adaptiveness depends on the stressor, the reliability of the parental and offspring environments and the evolutionary history of the population (Bell et al. 2016).

In addition to parental cues, exposure to environmental cues such as conspecific signalling or direct predator cues may provide juveniles with relevant information regarding the make-up of the forthcoming environment (DiRienzo et al. 2012). In rats, chronic stress during adolescence caused long-term changes both in foraging behaviour and foraging performance: under high-threat conditions, rats previously exposed to stress began foraging much sooner, made more transitions between foraging patches and consumed more rewards than previously unstressed rats (Chaby et al. 2015), indicating that early-life stress may be adaptive as it can enhance behavioural functioning in future high-threat environments. Evolutionary reasoning here is that when lack of food, unfavourable habitat or predation, sometimes collectively considered stress (see also “Stress” section), is experienced early in life, behavioural processes such as migratory, foraging and exploration behaviours need to be adjusted in order to maintain fitness later in life (Kasumovic and Andrade 2009).

Predictability of the later-life environment can be unreliable, causing behavioural adjustments that are mis-matched to future environments. The environment experienced in early-life may not match the environment experienced in later life for a variety of reasons, including environmental change, stochastic events and niche shifts, but also dispersal and migration (Burton and Metcalfe 2014). In addition, environmental cues are not always accurately perceived by parent or offspring (Paglianti et al. 2010; Bocedi et al. 2012). In order to optimise predictability of future environments, biological processes should have evolved to use as broad a sampling window and as diverse a range of cues as possible (Burton and Metcalfe 2014). It has been convincingly argued that a broader and more integrated life-history perspective is needed in order to understand the adaptive value of environmentally induced behavioural adjustments, taking into account both immediate and longer-term environmental context (Sheriff and Love 2013).

### Closer match to individual differences

A sometimes oversimplified cause for developmental sensitivity to early-life circumstances is that it allows animals to acquire useful coping behaviours fine-tuned to their personal characteristics. Even in (relatively) stable and unchanging environments, close and individualised adaptation to the environment is relevant. There are two main reasons for this: differential experience or impact and differential susceptibility or biological sensitivity to context. Although these are not new ideas (Rosenzweig and Bennett 1996), they have only recently been considered in terms of animal personality and coping (Ungar 2017).

The same early-life environment may be experienced differently by individuals based on life-history aspects such as birth order and birth weight (Biro and Stamps 2008), or any number of stochastic (life-history) events. In addition, the same environment may affect individuals differentially based on their innate susceptibility (Belsky and Pluess 2009b; Ellis et al. 2011; Jolicoeur-Martineau et al. 2017), as some individuals are more sensitive to negative effects of adversity as well as positive effects of opportunity (Pluess 2015). It has been suggested that there are also individual differences in underlying cognitive systems that are thought to facilitate individual’s capacity to plan effective coping behaviour, and that allow for the coping process to begin before a stressful event (Derryberry et al. 2003). Cognitive systems and life-history aspects, in turn, may differ due the impact of the early-life environment, causing complex interactions between the way individuals relate to their environment and are in turn impacted by it.

As a result, it is adaptive for animals to acquire behavioural patterns in early life that are adjusted for their unique individual circumstances, as shaped by the interplay between their phenotype, life history and personal early-life environment (Nettle and Bateson 2015). Individual differences in any number of such characteristics may necessitate non-genetic flexibility in the development of coping behaviours (Rödel et al. 2017) and lead to differences in the coping behaviours animals use throughout life (Wilson and Krause 2012). These differences often become consistent within individuals over the course of development (McGue et al. 1993; Derryberry et al. 2003; Bell and Stamps 2004; Caspi et al. 2005; Dall et al. 2012; Dochtermann and Dingemanse 2013).

Both differential experience and susceptibility link into the previously detailed precursors to coping (see “Coping behaviours: definitions and precursors” section): the amount of experience an animal has with a challenging situation, the extent to which it has seen examples of effective strategies, the degree to which it has practised those behavioural strategies and the resources it has available may be different even for its sibling growing up in the same habitat. It explains why individual differences in behaviour exist even for genetically identical twins growing up within a shared family environment (Asbury et al. 2003). A well-designed study on within-litter differences in rabbits (*Oryctolagus cuniculus*) showed that offspring consistently differed in postnatal body temperature and early growth. These individual differences in life history corresponded to consistent differences in coping behaviours: pups with lower body mass struggled more when handled and explored more in an open field test, while pups with higher body mass jumped sooner from a platform (Rödel et al. 2017). An earlier study showed that whether or not a rabbit becomes tame and relaxes, its stress behaviour when responding to humans is dependent on the state pups are in when exposed to human handling (Pongrácz and Altbäcker 1999). In cichlids (*N. pulcher*), a cooperatively breeding species of fish, rearing group size and the time juveniles spent in these groups affected the development of later-life social behaviours (Fischer et al. 2015). These empirical examples illustrate the importance of life-history aspects and other individual differences in the development of coping behaviours and suggest that early-life cues allow animals to develop coping behaviours better suited to their individual circumstances.

### Inheriting parental behaviours

Coping strategies may be transferred from one generation to the next without a genetic basis (Rosenzweig and Bennett 1996; Grether 2005; Leichty et al. 2012). Evolutionary theory suggests several advantages to non-genetic transmission of behaviour (Marshall and Uller 2007). Importantly, it allows learned behaviours to be passed on from one generation to the next. In reasonably stable and predictable environments, offspring gain a distinct advantage if their parents can pass on their own experience with current ecological conditions (Shea et al. 2011), and prime them for the situations they are likely to face in life. It allows juveniles to quickly obtain complicated responses and display behavioural strategies that proved successful to their parents, without having to take risks and gain experience with the environment themselves (see “Parents” section).

Another benefit of non-genomic transmission of behaviour concerns parent-child resemblance and parental investment: this theory is based on the understanding that males, unlike females, cannot be certain about paternity, and should provide less paternal investment to young who are unlikely to be their offspring (Bressan 2002; Apicella and Marlowe 2004; Anderson 2006; Heijkoop et al. 2009). It is expected for males to have developed ways to estimate relatedness through cues of physical and behavioural resemblance, and for offspring to have developed methods to increase resemblance to fathers also in cases where the father is not genetically related. For example, it was recently discovered through cross-fostering experiments in zebra finches that exploratory behaviour of foster parents, but not that of the genetic parents, was predictive of the exploratory behaviour of offspring (Schuett et al. 2013).

## How the early-life environment affects the development of coping/developmental processes affecting coping behaviours

Even though some of the evolutionary factors underlying coping are becoming clear (see “Why the early-life environment affects the development of coping” section (Øverli et al. 2007; Wolf et al. 2008; Hengartner 2017)), the developmental processes through which coping behaviours emerge have been studied to a much lesser degree (Stamps and Groothuis 2010; Groothuis and Trillmich 2011), and the mechanisms leading to coping behaviours later in life are largely unexplored (Haun et al. 2013; Miranda 2017). Here, we review the literature to shed light on the way coping behaviours develop, by categorising the developmental processes impacted by environmental factors. We discuss six important biological processes that affect development of coping behaviours during the early years of life: maternal effects, filial imprinting, habituation, conditioning and social learning (see Suppl. material). We illustrate the relevance of those developmental processes shown to affect later-life coping behaviours, and illustrate key differences between them in onset and development where possible. In this, we do not consider (epi)genetic effects as a separate process but rather acknowledge that epigenetics may well play a mechanistic role in each of the processes detailed below, the details of which are already explained excellently elsewhere (Weaver et al. 2004; McClelland et al. 2011; Cowan et al. 2016). For the same reason, we do not consider theoretical models, or empirical evidence of non-behavioural traits concerning these developmental processes. The most important processes were identified from the literature and discussed below.

### Parental effects

Maternal effects, defined as the direct effect of a mother’s phenotype on that of her offspring (Bernardo 1996; Reddon 2012), have been researched in detail both in animals and humans over the past decades. Developmentally, maternal effects relate to the need for developing systems to receive the appropriate (amount of) input. Depending on the quality and quantity of input received, the young develop or fail to develop a variety of phenotypic characteristics, including many coping behaviours. Maternal effects have been found across many species during the gestation period and after (Bernardo 1996; Mousseau and Fox 1998) and have been studied with respect to hormones (Adkins-Regan et al. 2013), nutrition (Langley-Evans et al. 2005), behaviour (Weaver et al. 2004), predator experience (Bell et al. 2016; Freinschlag and Schausberger 2016), birth weight (Taborsky 2006a) and maternal care (Champagne et al. 2003; Champagne and Meaney 2006), or a combination of all of the above. More recently, paternal effects have received more interest as well, although compared to maternal effects, still much less is known regarding the role of paternal factors (Rodgers et al. 2013). The most significant difference between maternal and paternal effects is the gestation period, during which the maternal phenotype is in intimate physical contact with that of the offspring, which allows for nourishment and hormones to pass from mother to child. Instead of separating between maternal and paternal effects, some studies consider the more generic parental effects (Uller 2008; Badyaev and Uller 2009; Burgess and Marshall 2014). The topic knows a rich literature of its own (reviewed in Badyaev and Uller 2009; Reddon 2012) that falls outside our scope to review in its entirety.

Early-life influences that affect offspring coping behaviours through parental effects can be as straightforward as parental behaviour, although this is underrepresented in empirical studies. Parents can change key parameters of offspring’s regulatory system through their behaviour prior to and after the offspring’s experience of a stressor and, in this way, change the offspring’s experience of the stressor and the way its developmental processes are impacted (Tang et al. 2014). Furthermore, cross-fostering experiments in zebra finches (*T. guttata*) showed that offspring exploratory type was predicted by exploratory type of the foster, but not the genetic parents (Schuett et al. 2013). In addition, the amount and quality of parental care seems to strongly affect the development of effective coping behaviours (Budaev et al. 1999; Moons et al. 2005). In convict cichlid (*Cichlasoma archocentrus*), parental activity during parental care was negatively correlated with the freezing versus activity factor in female offspring (Budaev et al. 1999). However, parental effects seem to be more complicated than a simple case of more care is better (Tang et al. 2014). The predictability of parental sensory signals has been shown to affect cognitive development, with unpredictable maternal signals leading to poor cognitive performance in behavioural tests (Davis et al. 2017). Beyond providing care and behavioural example, parental affects can prepare for fluctuating environments (Proulx and Teotónio 2017), mediate the impact of environmental input or stressors, as well as change the offspring’s experience of an environmental situation (Tang et al. 2014).

Especially maternal stress (Thornberry et al. 2009) and maternal care (see “Parents” section) have been shown to affect offspring coping behaviours (Champagne et al. 2003; Fish et al. 2004). It is becoming increasingly evident that maternal exposure to adversity during pregnancy can lead to life-long effects in offspring (Matthews and Phillips 2010). For example, male offspring of stressed rat mothers were more active in maze tests and entered the open arms of the maze more often than male offspring of control mothers (Götz and Stefanski 2007). The effects of maternal stress during pregnancy on behavioural outcomes in the first-generation offspring are thought to be highly dependent on species, sex and age (Sullivan et al. 2011), as well as on the time in pregnancy when stress is experienced (Matthews and Phillips 2010), although this is predominantly studied in rodents at the moment.

There has been some discussion whether the effects that parents have on their offspring’s phenotype are necessarily adaptive (Uller et al. 2013). Some maternal effects seem to have a clear adaptive advantage either for the mother, the offspring or both (Wells 2007a). For example, female western bluebirds (*Sialia mexicana*) increase androgen concentrations in their eggs as competition from their sister species increases, resulting in more aggressive male offspring that are more likely to disperse and create new colonies (Duckworth et al. 2015).While a body of experimental work implies adaptive advantages to parental effects (Mousseau and Fox 1998; Marshall and Uller 2007; Nätt et al. 2009; Jensen et al. 2014) and the term maternal programming is being used increasingly to indicate mother’s active preparation of offspring for future circumstances (Fish et al. 2004; Weaver et al. 2004; Langley-Evans et al. 2005), there are also indications that adaptive advantage cannot be assumed for all parental effects (Marshall and Uller 2007; Tang et al. 2014; Freinschlag and Schausberger 2016). For example, maternal exposure to predation risk actually decreases offspring anti-predator behaviour in three-spined sticklebacks (McGhee et al. 2012). It has been argued that the information a foetus receives is not in fact about the environment it is likely to face in its lifetime, but rather about the condition of its mother (Wells 2007b). Overall, the adaptive value of maternal effects is strongly ecologically dependent and can backfire under variable conditions. In such situations, parents may benefit by producing offspring that vary in sensitivity to particular experiences (Frankenhuis and Panchanathan 2011).

### Filial imprinting

Another process, which has often been overlooked since the initial interest in the 1960s (Bateson 1966), and which deserves much greater attention both in empirical work and theoretical study (Junco 2017), is filial imprinting. Related to the better studied sexual imprinting (Irwin and Price 1999; Witte and Sawka 2003; Kozak et al. 2011), filial imprinting is a process through which young individuals are capable of assimilating information and behavioural strategies necessary for their development (Hoffman and Ratner 1973), even when there is little information available or only for a short time. It has been most studied in bird species, as the preference of offspring to approach a stimulus to which they have been exposed early in their development (Bolhuis and Honey 1998), and an avoidance of dissimilar stimuli beyond normal avoidance of unfamiliar cues. For example, young male zebra finches preferred a song during which they were exposed during a sensitive period for song learning over their own song, or a new song (Adret 1993). Filial imprinting provides a means for information to be acquired at a time when sensory faculties have not yet developed, through processes different from learning (Ewer 1956), that seem to operate much earlier in ontogeny. Imprinting is expected to be especially relevant for those aspects of development that are sensitive to receiving the correct input on which to base development, for which there is a high cost of failure to receive correct input, and that concerns cues that occur early in the developmental process and are comparatively stable across evolutionary history (Remy 2010). It has been suggested that through imprinting, young can recognise their parents across a variety of conditions and respond appropriately to a particular posture or movement by a conspecific or predator which they have never seen before (Bateson 1966).

Filial imprinting is based on an ensemble of characteristics presented by the parents (Bolhuis and Honey 1998), rather than on a single attribute or stimulus, and can happen visually, auditory or entirely subconsciously (Bateson 1966; Batista et al. 2016). Some of the early work on imprinting indicates that imprinted preferences are surprisingly stable across an individual’s lifetime, even in the face of considerable experience with or even conscious training upon other stimuli (Ewer 1956; Bateson 1966; Salzen and Meyer 1968), and it has been known to affect offspring’s behaviour later in life, up to and including their mate choice (Witte and Sawka 2003; Bereczkei et al. 2004). Although the exact mechanisms through which juveniles imprint on their parents and others within the social group are still unclear, work in avian biology shows that juveniles are more likely to imprint on more conspicuous cues than less interesting stimuli (Bolhuis and Honey 1998). Great shock, among a number of factors, has been found to interfere either with the ability to imprint itself or with the coping behaviour given in response to imprinted stimuli (Bateson 1966), although there is also indication that increased stress during development should lead to individuals imprinting more strongly and rapidly (Kovach and Hess 1963). There is some suggestion that imprinting processes relate to sensitive periods in behavioural development (Knudsen 2004).

In the last few years, there has been an increasing interest in imprinting, specifically related to the development of behaviour. It is being considered as much more important to developmental processes than previously thought (Martinho and Kacelnik 2016; Santolin et al. 2016). Some consider imprinting a process similar to learning, responsible for early discriminatory abilities and social bonding (Junco 2017), or a process causing the young to be predisposed to social partners (Gyuris et al. 2010; Di Giorgio et al. 2017). However, it is becoming increasingly clear that imprinting does not only cause preferences that affect sociality. The process can also set preferences for more abstract concepts, such as preference for similarity or difference in a pair of objects (Martinho and Kacelnik 2016), preference for types of motion (Miura and Matsushima 2016), and even response to novelty (Versace et al. 2017). When chicks (*Gallus gallus*) were exposed to imprinting on similar or dissimilar items and through visual or acoustic modes or both, males showed more positive response to novel stimuli when they had been imprinted on dissimilar items. This response was even stronger when they had been imprinted across both modes. Females, on the other hand, were more attracted by familiar patterns (Versace et al. 2017). In that sense, it links closely to the aforementioned precursors to coping, perception (Di Giorgio et al. 2017, “Perception” section) and evaluation (“Evaluation” section). Recent evidence shows a close connection between imprinting and other developmental processes such as maternal care (Junco 2017) and relational concept learning (Martinho and Kacelnik 2016). For example, juvenile chickens showed a stronger preference for imprinted objects when they were being brooded or fed, suggesting that experiencing stimuli through usual maternal care is important for acquisition of imprinted information or preference (Junco 2017). Some success has been made using filial imprinting principles to affect the way domestic piglets cope with postweaning and crowding challenges (Mesarec et al. 2017). Although recent empirical work is lacking, it is likely that imprinting applies to behavioural cues as much as physiological cues, especially as imprinting has been shown to be sensitive to behavioural cues (Bolhuis and Honey 1998; Di Giorgio et al. 2017) and to similarity in coping strategies as well as auditory strategies.

### Habituation

Habituation is an important process through which individuals tune their coping behaviour to environmental cues, and one that is especially relevant during the developmental period. It is defined as a behavioural response decrement that results from repeated stimulation and that does not involve sensory adaptation/sensory fatigue or motor fatigue, and is commonly described by nine characteristics (described in Rankin et al. 2009). Practically, this means that animals tend not to give a startle response anymore when faced with environmental cues they have safely experienced many times before. Habituation to frequently occurring stimuli provides an evolutionary advantage as it shortens the time needed to evaluate a response, allows animals to disregard irrelevant repetitive stimuli (Caputi et al. 2016) and prevents unnecessary activation of defensive or aggressive behaviours. It is sometimes considered a prerequisite for other forms of learning and behavioural development as it allows animals to focus selectively on important stimuli (Rankin et al. 2009). In this, animals need to balance past experience against current threat levels (Hellström and Magnhagen 2017). Like filial imprinting, habituation is linked to parental care, and it has been shown that early-life stress by isolation disrupts habituation to external stimuli (Finamore and Port 2000).

Habituation has been studied especially within the context of response to novelty, as a confounder of experimental values of repeatability or exploration of novel situations. Individual differences in habituation are rarely studied (Martin and Réale 2008). However, rats reared in a social setting showed more rapid habituation to novel objects than rats reared in social isolation, which may account for higher exploration scores in isolated animals (Einon and Morgan 1976). Habituation of minnows (*Cyprinidae* sp*.*) to a predator cue was most rapid with the least realistic models (Magurran and Girling 1986), indicating a link between habituation and perceptual learning. More recently, differences in predator exposure during the first year of life in the Eurasian perch (*Perca fluviatilis*) were found to lead to differences in risk-taking behaviour even after being kept in a predator-free environment for 9 months (Hellström and Magnhagen 2017).

Processes of habituation are relevant especially in light of changing environments (Miranda 2017), where animals are often faced with repetitive environmental stimuli that they have no evolutionary experience with, and that they may not be able to estimate an appropriate coping response to (see “Coping behaviours: definitions and precursors” section). Animals who can successfully habituate to urban noise (Potvin 2017), light and other disturbances while still accurately estimating environmental threats have an important advantage in coping with change. This is illustrated by a study showing that urban house sparrows (*Passer domesticus*) habituated faster to urban disturbance than their rural conspecifics: while rural and urban birds were initially equally likely to hide, the urban birds came out of hiding faster over repeat trials (Vincze et al. 2016).

### Conditioning

Contrary to habituation, which occurs through simple repetition, behavioural conditioning occurs when a certain coping behavioural is consistently met with positive or negative reinforcement, through which the animal learns to perform one response and/or avoid another. For example, hatchery-reared rainbow trout (*Oncorhynchus mykiss*) that were conditioned to recognise chemical predator cues as dangerous, significantly increased anti-predator behaviours (decreased foraging, increased hiding), unlike trout from a control group. This response was still exhibited up to 21 days after conditioning (Brown and Smith 1998). Conditioning can work fast, when it concerns stimuli that are sufficiently harmful, but often works slowly if the negative reinforcement is not very consistent or if the payoff from taking the risk is higher than the cost of negative reinforcement (Adret 1993; Lovibond and Shanks 2002). As such, predictability of the response, both in quality and in timing, is essential in learning whether to avoid or approach a challenging situation (Krebs et al. 2017). For example, when mice were exposed to conditioned and unconditioned stimuli in unpredictable patterns, they began to express freezing behaviours even at the conditioned response (Seidenbecher et al. 2016).

Like other processes that allow for coping behaviours to be tuned to environmental conditions, conditioning appears to be especially effective early in life. In young male zebra finches (*T. guttata*), conditioning with a song as reward influenced the effectiveness of song learning during development but not song preferences in adulthood (Adret 1993). The effects of conditioning depend not only on age, but within that have also been shown to depend on state: within a small time window around nursing, rabbit pups (*O. cuniculus*) could be habituated to human handling, but not outside this window (Pongrácz and Altbäcker 1999). Similar to habituation, there are differences between species and individuals in the way conditions affect behaviour. For example, two closely related species of tadpoles (*Rana lessonae* and *R. esculenta*) were conditioned for 30 days to a variety of predators, after which species differences were found in the ways general activity levels and use of refuge changed, as well as differences in the type of predator they responded to (Semlitsch and Reyer 1992). Finally, again similar to habituation, behavioural conditioning depends strongly on the predictability of challenging situations and, as such, is relevant to changing environments, as animals often exhibit modulating behavioural changes (freeze, approach, etc.) preceding a predictable event they are conditioned to (Krebs et al. 2017).

### Perceptual learning

Perceptual learning is considered as any relatively permanent change of perception as a result of experience (Fahle 2004). This process allows individuals to distinguish between similar cues in their environment, for example to differentiate between a dangerous predator and a harmless animal (Brown et al. 2011) or to signal and perceive the identity of intra-group conspecifics (Rendall et al. 1996). Perceptual learning can occur not only under training conditions but also in situations of passive sensory stimulation, without awareness and without any task relevance (Watanabe et al. 2001; Seitz and Dinse 2007), which means that frequently occurring stimuli may sensitise perceptual systems, just as habituation desensitised them. It relates to threat and opportunity recognition (“Perception” section) and the ability to evaluate the correct response to environmental stimuli (“Evaluation” section), and subsequently can often affect adult behaviour (Beach and Jaynes 1954). Studies in humans have shown that perceptual learning can account for more than 76% of the rapid early improvement in performance (Hawkey et al. 2004).

Retention of perceptual learning is shaped by a suite of factors such as the strength of initial conditioning as well as individual personality. In a more recent empirical study, shy versus bold rainbow trout showed no difference in conditioned response, but there was a significant effect of personality on retention of learned predator recognition, where shy fish continued to display a conditioned response after 8 days but bold fish did not (Brown et al. 2013). In accord with this study, a low-responsive strain in the same species displayed longer retention of a conditioned response (Øverli et al. 2007). Recently, it has been argued that rather than having multiple perceptual systems, the animal-environment interaction cannot be correct unless individual forms of ambient energy such as light and sound exist in emergent, higher order patterns, i.e. a single overarching perceptual system that includes all kinds of perception (Stoffregen et al. 2017). This is relevant to the study of early perceptual learning, as empirical work is often (but not always) biased towards visual cues.

### Social learning

Social learning includes a wide range of mechanisms through which individuals receive and integrate information from other members of their social group. In animals, juveniles learn in very similar ways as humans, namely through mimicking the role model provided by conspecifics (most commonly parents), through active parenting by means of example and correction, and through conditional parenting based on offspring performance. What all such mechanisms have in common is that they involve learning from observation of a conspecific or from interaction with them (Heyes 1994; Hoppitt and Laland 2008). A special subset of social learning in humans and perhaps some kinds of primates is teaching, where older or more experienced members of the same social group intentionally pass on information, techniques or behaviours to juveniles. Through social learning, animals can acquire more information, skills and behaviours that allow them to deal with their environment than they might reasonably acquire based on personal experience, as shown for example in a study of wild meerkats (*Suricata suricatta*) where adults were shown to teach pups prey-handling skills (Thornton and McAuliffe 2006). Social learning also helps juveniles to learn the dominance hierarchy within a group, which facilitates group living and, as such, the protection and cooperation provided by a larger group size. A potential disadvantage is that individuals rely on others for the signal they get about environmental conditions rather than relying on their own sensory systems. Social learning is especially common in species where offspring are dependent on their parents for survival for a long time (van Schaik 2010), or where they live in strongly social groups. Mice, for example, are highly social animals, and young mice reared in a communal nest develop relevant social behaviours that mice reared with single mothers do not (Branchi 2009).

Models of social learning predict that animals living in stable environments should be more attentive to socially acquired information than animals living in variable environments (Zentall and Galef 1988), as social learning can be maladaptive when the information modelled by the parents has become outdated with regard to the current environment or when the environment is highly variable (Galef and Whiskin 2004; Laland 2004), causing juveniles to acquire coping behaviours that are mismatched to their environment (Lof et al. 2012). It can also be adaptive, as shown when fairy wren (*Malurus cyaneus*) learned to recognise unfamiliar sounds as alarm calls through social “eavesdropping” (Magrath et al. 2015). Models also predict that animals should be influenced more by the coping behaviour of older than younger group members (Benskin et al. 2002), as they are more experienced. Empirical work on rats supported the first, but not the second of these models (Galef and Whiskin 2004).

Through the development of behavioural patterns within social groups, rearing conditions can have lasting effects on the expression of adult coping behaviours (Rice 2008; Roulin et al. 2010). Of concern to conservation and coping with challenges of a changing world is recent evidence that wildlife can learn harmful behaviours from each other: whether or not bottlenose dolphins (*Tursiops aduncus*) become conditioned to being illegally fed by recreational fishers depended on both the degree to which the dolphins would frequent areas with a lot of tourism, and the degree to which they associated with previously conditioned dolphins (Donaldson et al. 2012). Not only is social learning in this way related to conditioning, it is also, like many of the other processes that link coping behaviours to the early-life environment, modulated by maternal care (Lindeyer et al. 2013).

## What in the early-life environment affects later-life coping behaviours

After reviewing why and how the early-life environment affects coping behaviours, we provide an overview of factors in the early-life environment that have been shown to trigger developmental processes to generate different coping behaviours. We discuss habitat, parents, nutrition, social environment, a selection of less studied influences and finally stress as an overarching influence. Various factors in early life may affect the developmental trajectory simultaneously (Rödel and Monclús 2011) and interact with the kind of experiences animals have during their life, the way they interact with conspecifics, as well as various life-history traits. It is expected that there will be a lot of variation both within (Belsky and Pluess 2009b) and between species in the extent to which environmental influences impact the different developmental systems. Furthermore, it should be noted that each of these influences likely impacts developmental processes differently depending on the sensitive window in development during which they are experienced (Fawcett and Frankenhuis 2015). However, given the structured and inherently canalising nature of developmental processes (Ellis and Boyce 2008), cross-species patterns are expected in the types of environmental influences that matter at different stages of development.

As epigenetics are likely to be involved in each of the developmental processes listed in the “How the early-life environment affects the development of coping/developmental processes affecting coping behaviours” section, stress appears to be a relevant detrimental influence across all of the categories below, whether it concerns stress from maternal separation (Biagini et al. 1998), lack of nutrition or unpredictable nutrition (Krause et al. 2009), bacterial infection or social difficulty. We briefly discuss stress in the “Stress” section.

### Habitat

Within eligible studies in this review (see Supplementary materials), effects of early-life habitat conditions were studied by means of natural habitat (Kelley et al. 2005; Moretz et al. 2007a; Aubret and Shine 2008; Sweeney et al. 2013), housing environment (Bolhuis et al. 2004, 2005, 2006), restricted bedding (Fuentes et al. 2014) and environmental enrichment (Roberts et al. 2011).

Most current work on the effects of early-life habitat on coping behaviours studies the question how laboratory and housing conditions affect animal’s coping style. Early-life housing conditions were extensively studied in pigs (*Sus domesticus*), where they were shown to affect coping behaviours: barren housed pigs were less active, less explorative and less playful and showed more social aggression than pigs raised in enriched housing (Bolhuis et al. 2005). Similarly, pigs from enriched housing were shown to have more difficulty responding to changes in a spatial discrimination maze than pigs from barren housing (Bolhuis et al. 2004), behaviour which relates to their ability to cope with changes in foraging conditions. Housing conditions did not affect all individuals equally: low-resisting pigs were more affected by adverse housing than high-resisting pigs across various behaviours, including chewing, manipulative and play behaviours (Bolhuis et al. 2006). In rats, behavioural effects due to restricted bedding during the first days after birth (in addition to another early life stressor) were shown to affect males and females differently across different coping behaviours (Fuentes et al. 2014). However, the effects of laboratory environment are by no means restricted to mammals. Laboratory-reared skiffia fish (*Skiffia multipunctata*) displayed increased courtship, aggression and curiosity towards a novel predator compared to pond-reared fish and commenced foraging on novel food more rapidly (Kelley et al. 2005). In juvenile Atlantic salmon (*Salmo salar*), exposure to enriched conditions improved learning and ability to navigate a maze without errors (Salvanes et al. 2013). Similarly, enrichment of rearing environment was found to affect several aspects of mahseer fish (*Tor putitora*) behaviour: exploratory behaviour, predation and anti-predation response were higher in fish reared in enriched or semi-natural environments than in barren environments (Ullah et al. 2017). Laboratory-reared spiders (penultimate *Agelenopsis pennsylvanica*) never exhibited a behavioural syndrome between boldness and foraging aggressiveness, while field-reared penultimates (but not juveniles) did (Sweeney et al. 2013).

In a more ecological setting, snakes (*Notechis scutatus*) were found to base habitat choice, an important life-history decision that affects coping success in many aspects of life, on the habitat type in which they had been reared—an adaptive effect as they were also found to be more effective in locomotion in these habitats than in others (Aubret and Shine 2008). There is some indication of effects of variation in temperature, humidity and wind speed, possibly modulated by resource availability, although this has not been studied specifically with regards to early-life environment (Love et al. 2013). While there is increasing interests in the effects of urbanisation, habitat fragmentation and quality on behavioural development, few studies have specifically considered how experiencing such habitats during early-life affects coping behaviours later in life.

It seems clear from the examples above that the early-life habitat has important effects on the development of coping behaviours, partially through the amount and quality of stimuli received, which impacts neurological development, and partially through a lack of experience with relevant environmental cues. Consequences of a suboptimal habitat include inability to survive in a natural setting, anxiety and aggressive behaviours, and difficulty in responding to novelty and change. Such effects of habitat and housing conditions experienced early in life are especially relevant with regards to experimental design of animal behaviour studies (Bolhuis et al. 2006; Trocino and Xiccato 2006) and the extent to which we can interpret coping behaviours from animals with non-ecologically valid early-life housing (Salvanes et al. 2013), but are also interesting in relation to an animal’s ability to adapt to shifts in habitat quality or size and to better understand the impact of urban conditions on previously rural species (Lapiedra et al. 2017; Miranda 2017).

### Parents

In many of the studies we reviewed, the role of the parents is extensive. Parents affect their offspring through genes, through the security and nutrition they provide, through the example they set, through their parenting behaviours such as grooming and licking, and the behaviour they actively encourage in their offspring through means of correction and approval. In addition, mothers affect their offspring through hormonal influences (Dufty Jr et al. 2002; Weaver et al. 2004; Champagne 2011; Giesing et al. 2011) during gestation. To an extent, and depending on the species, the environment the mother lives in actually creates the environment of her offspring (Duckworth et al. 2015). While some studies include parent-offspring interactions in their definition of social interactions, as juveniles learn important behavioural systems from both parents and others in their social group, for the purpose of this study, we consider them separate influences in order to clarify effects unique to the parent-offspring relationship. Within eligible studies, parenting was studied especially in the last decade in the context of maternal isolation or separation (Biagini et al. 1998; Caldji et al. 2000; Martin 2002; Ruedi-Bettschen et al. 2004; Moons et al. 2005) and, more recently, maternal stress (Champagne and Meaney 2006; Kiryanova et al. 2017; Rooke et al. 2017), which has often been related to intergenerational effects (see “Stable or stuck across generations?” section), and parental care and provisioning (van Oers et al. 2015).

Many studies found detrimental effects of maternal separation early in life. For example, rats (*Rattus rattus*) separated from their mothers early in life displayed reduced activity and risk taking, and increases in orienting time (Spivey et al. 2008), while other rats showed impaired coping (Ruedi-Bettschen et al. 2004). Calves (*Bos primigenius*) reared by mothers showed more escape and vigilance behaviour than automat-reared cows when faced with an isolation test, and displayed overall more social behaviours towards conspecifics (Wagner et al. 2013). In addition, chimpanzees (*Pan troglodytes*) separated from their mothers and/or social group as juveniles showed decreased activity and increased abnormal behaviours, with stronger effects in younger individuals (Martin 2002). Other studies found an effect of early postnatal handling but not of maternal separation on the ability of juvenile rats to cope with novelty (Biagini et al. 1998), and adult rats who had been handled as juveniles showed reduced startle responsivity, increased exploration and decreased suppression of feeding, whereas those that were maternally separated as juveniles did not (Caldji et al. 2000). Such effects of handling (see also Núñez et al. 1996; Spivey et al. 2008) indicate that separation and handling cannot be considered comparable influences, and call for caution when studying the effects of parental separation. In addition, some studies have considered more complex systems and interactions between life history and parental behaviour on the young. For example, in Japanese quail (*C. japonica*), mothers of large broods emitted more maternal vocalisations at the start of mothering but covered the young less towards the end. Chicks in turn huddled more and had higher social motivation than chicks in small broods (Aigueperse et al. 2017). Impaired maternal care (Champagne et al. 2003; Lindeyer et al. 2013), which may in turn be caused by poor habitat conditions (Ivy et al. 2008), and maternal stress (Rooke et al. 2017) have also been empirically linked to the development of coping behaviours.

Beyond maternal stress, care and separation, some studies have looked at parental behavioural traits in relation to offspring coping, showing that juvenile cats (*Felis catus*) from friendly fathers were quicker to approach, touch and rub an unfamiliar person and remain in close contact with novel objects than those from unfriendly fathers (McCune 1995). In contrast, offspring of exploratory zebrafish females were always highly exploratory regardless of behavioural traits of the father (Wisenden et al. 2011). Such similarities cannot blindly be ascribed to genetics. In zebra finches (*T. guttata*), offspring exploratory type was predicted by the exploratory behaviour of the foster, but not the genetic parents (Schuett et al. 2013).

Finally, parental experience has been shown to affect offspring coping behaviours. In lizards (*Zootoca vivipara*), offspring of mothers exposed to predator cues dispersed three times more in an unfamiliar semi-natural environment than offspring from unexposed mothers (Bestion et al. 2014). In sticklebacks (*Gasterosteus aculeatus*), females exposed to predator cues produced offspring with altered behaviour, and males (who provide parental care) exposed to predator cues produced offspring that were less active (Bell et al. 2016). Not only predatory experience but also social experience of parents can be a relevant factor. In wild cavies (*Cavia porcellus*), an unstable social environment experienced by mothers during pregnancy and lactation led to camouflage behaviours in male offspring, whereas a stable social environment led to earlier reproductive behaviour (Siegeler et al. 2017). No differences were found between coping behaviours of cavies whose mothers experienced a socially unstable environment compared to those who experienced stable social conditions (Kemme et al. 2008). It should be emphasised that parental experience and parental stress are often overlapping factors in the studies reviewed here, and that while it is often assumed that parental exposure to predation constitutes as stress, it may have ecological implications beyond stress especially with regards to parental effects on offspring coping behaviours.

### Nutrition

Nutrition is perhaps the most important building block for development of the physical body. More recently, it has also been linked to the development of behaviour (Noguera et al. 2015; van Oers et al. 2015), as lack of appropriate nutrition affects the mechanics of developmental systems, potentially stunting or derailing healthy development. Several studies examined the effects of yolk reserves (Andersson and Hoglund 2012), food availability (Carere et al. 2005; Edenbrow and Croft 2011), food quality (Krause et al. 2009; Tremmel and Müller 2013) and body weight (Rödel and Monclús 2011) on coping behaviours and found that effects depend strongly on species and an interplay of surrounding factors.

Exploration and activity are behaviours often studied in relation to early-life nutrition. Female zebra finches raised on low-quality food were faster to show exploration and foraging behaviours than those raised on high-quality food, although there were no differences in latency to move after the start of the experiments (Krause et al. 2009). Similarly, low availability of dietary micronutrients during the postnatal period was found to lead to reduced boldness in male zebra finches, but did not affect fear of novelty and did not affect females at all (Noguera et al. 2015). In European rabbits (*O. cuniculus*), exploratory behaviour and anxiety levels were correlated with body mass in early life, but not with body mass and age at the time of testing (Rödel and Monclús 2011). In a species of beetle (*Phaedon cochlaeriae*), low-quality food induced boldness, and by extension, potential foraging success, while animals raised on high-quality food were more active (Tremmel and Müller 2013).

In addition, many studies found that food conditions during early-life affected later-life aggression. Young trouts (*O. mykiss*) with larger yolks showed more aggressive personality traits than siblings with smaller yolks and, subsequently, were more aggressive in territory establishment and more socially dominant (Andersson and Hoglund 2012). However, in mangrove killifish (*Kryptolebias marmoratus*), low food conditions reduced exploration but not boldness or aggression (Edenbrow and Croft 2011). In a selection line of great tits (*Parus major*) selected for fast exploration, food rationing was linked to increased aggression (Carere et al. 2005), yet in a different line of great tits, juveniles provisioned with less food exhibited a stronger stress response and faster exploration later in life than juveniles provisioned with more food (van Oers et al. 2015). Bulb mites (*Rhyzoglyphus* sp*.*) raised on rich diets developed an aggressive phenotype, whereas those raised on poor nutrition became non-aggressive “scramblers” (Smallegange 2011).

These studies indicate that the effects of nutrition do influence adult coping behaviours across many different species, but nutrition does not necessarily affect all behaviours, or both sexes, or in the same way. It has been argued that the quantity, quality and predictability of resources can act as ecological stressors in mothers (Love et al. 2013) and, as such, affect their offspring through various developmental pathways. Negative consequences of food deprivation during ontogeny may reappear especially when environmental conditions deteriorate in adulthood (Krause et al. 2009).

### Social group

The relationship between individuals and a group of conspecifics is often referred to as their social environment or social group, which especially in recent years has been an increasingly studied topic in animal behaviour. The social group is important in processes of social learning (see “Social learning” section) but also relates to safety from predators for animals living in groups, competition for resources and dominance interactions (Love et al. 2013). Within selected studies, the effects of early-life social environment were studied quantitatively in the context of early-life social isolation (Van Den Berg et al. 1999; Tuchscherer et al. 2006; Kemme et al. 2008; Tanaka et al. 2010; Hesse et al. 2016; Riley et al. 2017) and group size (Edenbrow and Croft 2011; Naguib et al. 2011; Rödel and Meyer 2011; Niemelä et al. 2012; Riley et al. 2017), and qualitatively in the context of relationships with siblings and other conspecifics (Carere et al. 2005; Moretz et al. 2007a; Hudson et al. 2011; Wismer et al. 2014). The effects of social group are predominantly studied in rodents, specifically rats. Frequency, duration and latency of various social behavioural elements such as social exploration, approach/following and anogenital sniffing were affected when juvenile rats were isolated from their social group. However, once social contact was initiated, a relatively normal behavioural pattern was displayed (Van Den Berg et al. 1999). Other studies found that socially isolated juvenile rats had difficulty with social recognition (Tanaka et al. 2010). In rodent species like rats and mice, social environment can be studied through the communal nest, an experimental setting similar to a natural nest, in which juveniles can interact with peers as well as with their mother and siblings (Branchi 2009). Communally reared rats were found to display reduced anxiety-like behaviour when exposed to novel environments, and females were more subordinate and less aggressive when exposed to an intruder male (Curley et al. 2009).

The influence of the presence and absence of a social group has also been studied in other species. Group housing in zebra finches (*T. guttata*), for example, was related to a higher number of social interactions in males (Bölting and von Engelhardt 2017). In cichlids (*N. pulcher*), fry raised with adults showed more aggressive and submissive behaviour to each other than fish raised with siblings only (Arnold and Taborsky 2010). When early-life social rearing was combined with simulated predation threat in cichlids, individuals developed specialised behavioural competences leading to different developmental trajectories with respects to, among others, dispersal (Fischer et al. 2017). In addition, a study on water striders (Gerridae sp*.*) showed that being raised in a social environment affected the degree of behavioural plasticity males exhibited later in life, i.e. the flexibility they had to choose a coping behaviour depending on the situation at hand, something that can significantly affect a male’s fitness (Han and Brooks 2014). Spiders (*Marpissa muscosa*) reared in groups are better at exploration of a maze and at sociality than those reared in isolation, which is especially relevant since isolated rearing adds no known additional stress in this species (Liedtke and Schneider 2017).

Not only the presence or absence of the group was found to be relevant to behavioural development, but also size of the group. Litter size affected anxiety in rats in a non-linear way: young rats born to small- or large-sized litters had higher scores of anxiety-like behaviours than those from medium-sized litters (Rödel and Meyer 2011). A review on rabbits, rats and mice showed that early sibling relations, such as position in the huddle, contributed to the development of individual differences in behavioural style (Hudson et al. 2011). Group size also affected aggression, fear and display behaviours in black-headed gulls (*Larus ridibundus*), which was not reversed after birds were re-housed in larger groups (Groothuis and Mulekom 1991). Rearing-group size and the time juveniles spent in these groups interactively influenced the development of social skills in cichlids (Fischer et al. 2015).

Social group appears to not affect the development of all coping behaviours, even in species that are sensitive to social cues during development, nor does it necessarily affect sociality itself. An interesting study into the effects of housing different strains of zebrafish (*Danio rerio*) together as juveniles showed that the type of social experience during early life affected social behaviours: all mixed-group fish were more inclined to biting, while mixed-group fish of one strain were also more exploratory. No effects were found on activity, predator response or stress recovery (Moretz et al. 2007b). In field crickets (*G. integer*), young males reared in the presence of conspecific acoustic sexual signals were less aggressive and less likely to become dominant relative to those reared in the absence of acoustic signals (DiRienzo et al. 2012). Studies such as these show that there is a need to look beyond simple presence/absence of social group when studying sociality as an early-life factor impacting coping behaviours.

### Other influences

There are many environmental influences beyond habitat, parents, nutrition and social group, which affect the developmental processes leading to coping behaviours. Among them, predation is perhaps the most studied due to its evolutionary relevance, although it is unclear whether it is also the most relevant to development. The lesser studied early-life influences appear to be very relevant in affecting development of coping behaviours, either by affecting the coping behaviour directly or by affecting the degree to which coping behaviours are canalised into behavioural syndromes or animal personalities.

Predation is considered a very strong selection pressure. Predation can have a very strong impact on behavioural processes. Even a single cat encounter was shown to cause lasting increases in anxious behaviour in laboratory rats (Adamec and Shallow 1993). The effects of predator cues are often studied in relation to behavioural syndromes consisting of multiple coping behaviours (Bell and Sih 2007; Brown et al. 2013). Tadpoles (*Rana dalmatina*) reared alone and without predatory cues showed no personality and no behavioural syndrome between activity and risk-taking behaviours, while those raised with predator cues displayed activity and risk-taking personality traits, and those raised with predators and conspecifics both displayed an activity—risk-taking behavioural syndrome (Urszán et al. 2015). The effects of predation are neither consistent nor predictable. Wild-caught guppies (*Poecilia reticulata*) born and raised in high predation localities showed more boldness than those from low predation localities (Harris et al. 2010), rather than less. Understanding the effects of predation on behavioural development and coping is increasingly relevant in relation to human disturbance of natural populations, as to a certain extent, human-caused disturbance can be experienced by animals as a kind of predation (Frid and Dill 2002).

Increasingly, immunology is also considered a relevant early-life influence on behavioural development. In mallard ducks (*Anas platyrhynchos*), non-pathogenic immune challenges, administered at different stages of development, were found to affect both activity and exploratory behaviour, as well as colour-dependent novel object exploration. Specifically, individuals immune-challenged during mid and late developmental stages were more active in novel environments (Butler et al. 2012). In mice, pups born through C-section showed more anxiety behaviours in later life than those born vaginally, presumably through changes in the microbiota gut-brain axis (Morais et al. 2017). Similarly, neonatal exposure to environmental bacteria was shown to alter the development of anxiety-like behaviours in Fischer-344 rats, which expressed during adulthood but not during adolescence (Walker et al. 2004). In field crickets (*G. integer*), early pathogen exposure did not influence mean boldness behaviour directly, but it did affect the presence of a personality construct (DiRienzo et al. 2015).

Other influences include early-life cognitive stimulation, which in case of the red junglefowl (*G. gallus*) altered later-life personality traits of vigilance and escape attempts (Zidar et al. 2017) and in case of dogs decreased fear of novel objects (Pluijmakers et al. 2010). Photoperiod, especially in tandem with birth weight and size rank in the litter, was shown to affect exploration and boldness, in that where heavy females born in spring were more bold and explorative, but heavy females born in autumn were less explorative. Lighter females showed no such difference (Guenther and Trillmich 2013). Related to this, it has been suggested that differences in photoperiod and acoustic environment were the cause for conflicting results between studies into zebra finches’ (*T. guttata*) social behaviour (Bölting and von Engelhardt 2017). In dogs, the largest environmental factor associated with separation anxiety and stress sensitivity to noise was the amount of daily exercise during early life (Tiira and Lohi 2015).There is a growing interest in human-influenced effects on behavioural development, often in the context of global change, on topics including human disturbance, ecological stressors, temperature and climate variability, several of which are reviewed in Love et al. (2013). Some human-influenced early-life factors that were shown to affect coping behaviours include pollution effects like lower visibility or nutritional richness (Krause et al. 2009).

### Stress

There is overwhelming empirical evidence that negative early environmental influences impact adult behaviour (Blanchard et al. 2001; O’Mahony et al. 2009; Enoch et al. 2010; McClelland et al. 2011; Burton and Metcalfe 2014; Bolton et al. 2017). The effects of such negative influences are often clustered as “stress”. Rather than a separate type of early-life influence, stress represents unfavourable conditions beyond what the animal’s biology can easily cope with and can occur in many variations (Moberg 1985; Belsky and Pluess 2009b; McClelland et al. 2011). Stress during early life can cause disease and cognitive impairment but has been also argued to program developmental pathways (Farine et al. 2015; see also “Preparing for hardship” section). Stress experienced by the previous generation can produce profound and long-lasting perturbations of individual adaptive capacities, as shown in prenatally stressed rats who displayed impaired social play behaviour, enhanced anxiety and increased stress reactivity (Morley-Fletcher et al. 2003).

It would be too short-sighted to see early-life stress only as a detrimental factor. In a very inclusive review on the effects of early-life stress in squirrel monkeys (*Saimiri sciureus*), it is described how brief intermittent exposure to early life stress (through short maternal separation) actually promoted the development of arousal regulation and resilience, rather than leading to vulnerability (Lyons et al. 2010; Beehner and Bergman 2017). At 9 months of age, previously separated monkeys showed fewer signs of anxiety and increased exploration compared to non-separated monkeys, and at 2.5 years of age, they exhibited more curiosity in stress-free situations. These differences could not be ascribed to maternal mediation or long-lasting changes in maternal care following separation (Parker et al. 2007). However, very similar experiments in rats showed phenotypic changes including increased anxiety and health problems (O’Mahony et al. 2009). Rats who were exposed to chronic social, physical and predation stress in adolescence were slower to explore a foraging patch in later life under low threat, but also obtained more rewards under high threat (Chaby et al. 2015).

Examples such as these illustrate that animal behavioural development is complex, and operates as a well-tuned system to adjust behaviour to the experienced environment both when that environment is favourable, and when it causes stress. Findings on male Balb/c strain mice, for example, suggested that exposure to adverse early-life conditions did not make mice more vulnerable to later-life stress, but rather prepared them to better cope with a challenging adult environment (Santarelli et al. 2017). As most empirical studies consider stress through maternal separation in mammals, there is insufficient comparative material to indicate how stress in any of the other main environmental influences would impact development differentially. Some theoretical models regarding early-life stress on behavioural syndromes are given in Sih (2011)).

## Stable or stuck across generations?

Perhaps one of the most important environmental challenges for animals to cope with in our current world is change. Changes in the environment are especially unsettling because they require animals to respond in new, different ways, with no guarantee that the response they select will be functional. While developmental processes (see “How the early-life environment affects the development of coping/developmental processes affecting coping behaviours” section) have formed over evolutionary time through survival of those young animals who most effectively adjusted their coping behaviours to environmental cues, such processes may suddenly be disadvantageous under changing conditions. There is growing evidence from natural systems for intergenerational effects of early-life conditions (Berman 1990; Maestripieri 2005; Matthews and Phillips 2010; Zimmer et al. 2017), where environmental experiences during ontogeny of one generation affect the expression of phenotypes in the next generation (Champagne 2010; Moran et al. 2010; Cowan et al. 2016). Such effects can be generated by multiple environmental cues, affect offspring in many ways and can be transmitted directly or indirectly by both parental lines for several generations (Burton and Metcalfe 2014). Through intergenerational transmission, once-adaptive robust developmental processes may cause offspring to be adapted to environmental conditions of several generations ago rather than their current or predicted future environment. In addition, developmental processes for many animal species may not be equipped to acquire accurate information about or under novel environmental conditions, such as street lights and urban noise (Miranda et al. 2013). Given the strong effects of the early-life environment to shape a multitude of behaviours and coping skills later in life, we have to ask the question: in our current radically and fast changing environment, do our early life experiences help to create stable behaviours and coping skills, or do they leave individuals stuck in the past?

Here, we discuss literature pertaining to the extent to which environmental effects on the development of coping behaviours allow for a stable adaptation to the environment, with the flexibility to adjust to change, or alternatively make animals vulnerable to unfavourable traits inherited from previous generations. In the literature, there exists a confusing overlap in terminology between parental effects (see “Parental effects” section) and intergenerational effects. To mediate this confusion, we define intergenerational transmission as non-genomic effects in which a particular phenotypic expression is transmitted from one generation to the next, whereas maternal effects can be considered simply the effects of maternal phenotype on offspring phenotype. Following this, to borrow an example from studies on humans, cases where offspring experience behavioural disorders linked to maternal smoking during pregnancy (Abbott and Winzer-Serhan 2012) constitute maternal effects, while cases where offspring suffer from a smoking addiction due to maternal smoking during pregnancy (Hellström-Lindahl and Nordberg 2002) should be considered intergenerational transmission of coping.

### Flexible adaptations

One of the most-named advantages of developmental sensitivity to early-life environments is that it allows animals to generate phenotypes more likely to be adaptive, regardless of the genetic blueprint. One of the ways in which this has been demonstrated is through mediation of previous negative effects, thus re-adjusting developmental processes that may have become derailed by adversity. The early-life environment seems to have a capacity to normalise behavioural dysfunctions produced by prenatal stress, at least in some situations. Juvenile prenatally stressed rats showed reduced play behaviour and increased emotionality. Enriched housing increased the amount of time these juveniles engaged in positive species-typical behaviour and reduced emotionality, and mediated induced immune challenges (Laviola et al. 2004).

Recently, with the rise in studies into global change, intergenerational effects have been mentioned specifically as a mechanism through which animals can adjust to fast changing and novel environments that their genetic make-up, as a product of natural selection, has not prepared them for (Meylan et al. 2012; Miranda et al. 2013; Lapiedra et al. 2017; Spadafora 2017), although such effects might be rarer than commonly thought (Uller et al. 2013). This may be particularly relevant in situations where animals have to adapt their coping behaviours quickly to evolutionary novel environments, as found in the move from rural to urban habitats (Vincze et al. 2016; Miranda 2017). For example, lizards (*Anolis sagrei*) from a population in urban areas were more tolerant of humans, less aggressive and bolder after a simulated predator attack and spent more time exploring new environment than lizards from a population in a nearby forest. These changes in behaviour were suggested to be due to adaptive behavioural adaptations to novel selective regimes (Lapiedra et al. 2017).

### Preparing for hardship

In contrast with prevailing views that stress effects are cumulative and increase stress vulnerability throughout life (Santarelli et al. 2017), many recent studies have begun to suggest that the effects of early-life environment may help buffer later-life hardship. By receiving cues early in life that the environment in adulthood is likely to be difficult, animals have an opportunity to adjust their development towards coping behaviours that are more likely to be successful. For example, male Balb/c strain mice were exposed to adverse and supportive early-life conditions, and then given a socially adverse or supportive environment in adulthood. Negative consequences for stress responsiveness and anxiety-related coping behaviours were found mainly in mice that were exposed to either early-life or adult adversity, but not in mice that were exposed to both (Santarelli et al. 2017). Another study in mice found that early-life stress through maternal separation improved coping with and recovery from a traumatic social experience in adulthood (Zoicas and Neumann 2016).

Preparatory effects of the early-life environment are not limited to stress. Juvenile cinnamon anemone fish (*Amphiprion melanopus*) were less reactive and had poorer locomotive performance when exposed to elevated CO_2_ levels, but parental exposure to high CO_2_ reduced these effects in some—but not all—traits (Allan et al. 2014).

### Passing on the past

There is increasing evidence that environmental effects experienced even before conception can be transmissible to subsequent generations (Burton and Metcalfe 2014). There are several cases, notably in rodents, in which unfavourable conditions in previous generations negatively impacted coping behaviours of current and future generations. For example, dams that were exposed to chronic social stress displayed impaired maternal care to their first generation (F1) offspring, who in turn displayed impaired maternal care to their (F2) offspring. Both male and female offspring displayed decreased social behaviour (Babb et al. 2014). Hypo-responsiveness to stress across several behavioural categories was shown in rhesus macaques (*Macaca mulatta*) whose mothers had been exposed to early-life stress, even when those mothers no longer displayed hypo-responsiveness themselves (Kinnally et al. 2013). Similar patterns were found in Japanese quail (*C. japonica*), where prenatal stress experienced by mothers resulted in the same stress-coping traits in the offspring across all phenotypic levels studied, including behavioural traits (Zimmer et al. 2017). Abusive behaviour as well can be transmitted across generations: more than half of the female rhesus monkeys who were abused by their mothers during their first month of life displayed abusive parenting with their firstborn offspring, regardless of whether they were reared by their biological mother or a foster mother (Maestripieri 2005).

A recent term for negative effects from multiple generations ago is ancestral stress or ancestral trauma (McCreary et al. 2016; Ambeskovic et al. 2017; Faraji et al. 2017), to differentiate from single-generation parental effects. There is some indication that ancestral trauma can be advantageous to a certain extent, but causes greater vulnerability to additional stressors later in life. In rats, a family history of recurrent ancestral prenatal stress was related to improved movement and skilled reaching, relevant in foraging behaviours, compared to rats without ancestral stress. The advantage of ancestral stress disappeared when rats experienced another stressor in adulthood, suggesting multiple stressors may limit behavioural flexibility rather than improve it (Faraji et al. 2017), even in a system where ancestral stress leads to adaptive developmental adjustments. In similar studies, social isolation during later-life stress caused cognitive retardation and reduced stress coping in rats from ancestral stress lines, but not in rats from unstressed lines (Faraji et al. 2017). In mice, postnatal trauma of unpredictable maternal separation and maternal stress altered coping behaviours in adverse conditions both in males when adult, and in their adult male offspring. These behavioural symptoms were not transmitted to the next generation when fathers experienced an enriched environment (Gapp et al. 2016).

It is not only negative experience that is passed on through intergenerational effects, however. Mouse dams rearing pups in communal nests displayed increased maternal care, to which their F1 offspring displayed reduced anxiety-like behaviour when placed in a novel environment, and increased quality of parenting behaviours towards their own offspring. F2 offspring also displayed reduced anxiety-like behaviour and better parenting compared to mice raised outside a communal nest (Curley et al. 2009). An elegant early study in vervet monkeys (*Cercopithecus aethiops sabaeus*) showed that the amount of contact mothers had with their offspring was related to the amount of contact they themselves had with their mothers. In this, a female’s experience in infancy was a better predictor of adult mothering than variables such as social learning as a juvenile, shared circumstances and average similarity between mothers and daughters (Fairbanks 1989).

The effects of (negative) environmental cues across multiple generations have been studied almost exclusively in rodents and are often targeted to understanding depression, anxiety and effects of stress in humans. This one-sided approach makes it more difficult to place empirical findings in an ecologically relevant context, although it does provide advantages in testing the limits of the developmental system in ways ecological studies in the wild would not. It might be argued, given the positive effects on coping behaviours consistently shown by cage enrichment and communal housing, that laboratory conditions in themselves represent multigenerational environmental stress on the level of habitat, parents, social group and possibly other influences.

### Resilience and reversibility

Important concepts when discussing the adaptive advantages and disadvantages that come with sensitivity of developmental processes to early-life conditions are resilience and reversibility. Resilience, the capacity to recover from difficulties, is important in estimating the strength with which negative experiences continue to influence animal health and fitness (Walker et al. 2006; Cicchetti 2010). Reversibility relates to the ability to reverse or undo the effects of (harmful) environmental conditions (Francis et al. 2002; Ruedi-Bettschen et al. 2004), and is especially important once coping behaviours have been established yet found unfavourable, or when the conditions on which their development was based have changed. While adaptation to early-life conditions can clearly be an adaptive mechanism, the ability to reverse the effects of early-life environments seems equally adaptive especially in the case of improved conditions or changed environments from infancy to adulthood.

Although some negative influences of the early-life environment have been shown to be reversible, this reversibility does not appear to apply to all affected coping behaviours. While there is evidence that beneficial later-life circumstances can partially remedy or even completely negate developmental limitations (Salzen and Meyer 1968; Francis et al. 2002; Gabriel 2005), there is also evidence that negative experiences during development continue to negatively affect behavioural functioning for the entire lifespan of an individual (Champagne 2010). For example, the effects of prenatal stress have been shown to be reversible by enrichment of the physical environment. Cage enrichment during pre-adolescence increased social behaviour and reduced response to stress in prenatally stressed rats, while the same enrichment showed no effects on non-stressed rats (Morley-Fletcher et al. 2003). In part, this may be linked to the timing of the negative influence (Keiley et al. 2001; Knudsen 2004).

Reversibility of early-life damage such as stress experienced by either mother or child has been shown in mice (Curley et al. 2009), rats (Champagne et al. 2003; Cui et al. 2006) and humans (Keay and Bandler 2001; Francis et al. 2002). Reversibility of imprinting on early-life stimuli, however, seems to be much harder as imprinted behaviours appear quite stable throughout later in life even with extensive training upon other stimuli (Salzen and Meyer 1968). As the processes of early perceptual learning, habituation and conditioning as such have not received much attention, little is known as to the differences between these processes in resilience and reversibility. However, the reversibility of the effects that social learning has on coping behaviours has been studied to a greater extent, and the effects of maternal separation and social deprivation have been shown to be reversible in some species and some situations. For example, for young chimpanzees who grew up without their mother or without conspecifics at all, recovery of healthy behaviours may occur with access to an enriched social environment (Martin 2002).

In some cases, especially when environmental conditions impact developmental processes that occur early in ontogeny or canalisation, negative behavioural consequences of environmental influences may not be reversible. In such a case, the individual finds itself stuck with the coping strategies developed during early life and may suffer fitness consequences. For example, cross-over studies in animals suggest that exposure to adversity in early life does not necessarily increase fitness in tough adult environments. Rather, those malnourished in early life do worse in adult environments, but particularly so in tough adult conditions (Taborsky 2006b).

In other cases, however, the effects of early-life conditions, even when they have been carried over across many generations, may still be reversed. For example, the Balb/c mouse strain is often considered “socially-incompetent” and anxious, yet when the young of this strain were reared in a communal nest instead of under regular laboratory conditions, anxiety-like behaviours and parental care were improved in both first- and second-generation offspring (Curley et al. 2009). Results such as these imply that the harmful coping behaviours in this strain may be a result of intergenerational effects of standard housing conditions, which can be attenuated both within and across generations in cases where the appropriate environmental cues allow the harmful coping behaviours to become reversible. This example from animal models is encouraging and may be applied in other species, including humans.

## Discussion

As previously indicated by several studies (Stamps and Groothuis 2010; Gracceva et al. 2011; Groothuis and Trillmich 2011), the relationship between external events and individual’s resulting coping behaviours has been surprisingly understudied. These statements are corroborated by our review, which yielded comparatively few papers that specifically addressed developmental processes, and the majority of those addressed epigenetic mechanisms and parental effects (see Supplementary material). The reason for the lack of a framework for developmental processes becomes clearer when reviewing the large number of coping behaviours studied in the literature and the diversity of early-life conditions: a multitude of behavioural aspects, the correlations between which are still poorly understood, are influenced by an equally large number of environmental conditions in sometimes opposite directions, with important differences between and within species, genders and even individuals. As different fields of study each has its preferred study species, environmental conditions and coping behaviours, comparing the available studies and constructing a bigger picture poses a challenge. Especially for that reason, however, there is a need to study and uncover the pathways through which early-life condition affects coping, so that a linear “this condition leads to that expression” can be replaced with a deeper understanding of the development of behavioural patterns.

Although of course the mechanisms leading to intergenerational transfer of behaviours and coping traits consist of a complex interplay between ecological and physiological factors (Wells 2011), of the processes linking early-life environment to development as discussed in the section “How the early-life environment affects the development of coping/developmental processes affecting coping behaviours”, maternal effects and imprinting are likely to be the most important in generating intergenerational effects. And indeed, we find many examples in the literature of cases where such processes affect coping in juveniles (Mousseau and Fox 1998; Agrawal et al. 2001; Marshall and Uller 2007; Moran et al. 2010; Champagne 2011), although more attention has been paid to maternal effects than to imprinting (Champagne 2011; Drake et al. 2011). The early-life influences responsible, following recent literature, are most likely to be social and reproductive behaviour and stressors experienced in these areas (Champagne 2010), although there appears to be somewhat of a bias in the literature towards studying mainly maternal influences (Champagne and Meaney 2006) on offspring social and emotional behaviour, and mainly in rodents and primates.

We recommend more targeted studies that focus less on relating an environmental influence to a behavioural expression, and more on the functioning of processes through which individuals ascertain and integrate external information, and in turn translate this information to behavioural patterns. Individual developmental processes, such as maternal effects, imprinting, habituation, conditioning or social learning, have been well studied for decades across fields of animal ecology, neurology and psychology, but with the exception of maternal effects, have not been extensively studied in the context of the development of coping behaviours. In relation to this, we suggest a move towards understanding the complex interactions between life history and development of coping behaviour. Nutrition, for example, tends to relate to body weight, which in turn has been shown to affect boldness (Mayer et al. 2016). Increased nutrition, however, can relate to better habitat, position in the nest, quality of maternal care, offspring’s own behaviour or any combination of these. These interactions between life history and developmental processes are complicated further by individual differences in sensitivity and experience, which cause differences in coping behaviours between animals that make us question potentially all of behavioural work. Differential susceptibility and developmental causes for such susceptibility may provide invaluable insights into the evolutionary reasons for early-life effects on the development of coping behaviours.

Furthermore, there is a need to understand parental effects and stress under natural or at least semi-natural conditions, and to begin to re-interpret findings from lab studies on behaviour in their ecological context, as behaviours from developmentally disturbed animals. We expect that such an approach will shed light on inconsistencies currently reported in this field (Moons et al. 2005), especially relating to parental effects and stress, as interactions of developmental system become better understood. A clearer understanding of more complex interactions will be especially useful in relation to resilience and reversibility of unfavourable traits, both of which are becoming increasingly important at the moment as most species, including humans, are challenged with large amounts of environmental change.

We call for more cross-fertilisation between different fields with regards to understanding how early-life influences impact animals’ ability to cope with their environment, a topic that is becoming increasingly important in behavioural ecology, conservation, under the topic Evo-Eco-Devo, as well as animal husbandry. It is clear that there are preferences within different fields to study certain factors of interest only in certain species, which inevitably causes a bias in our understanding of the various early-life influences and makes it difficult to consider ontogeny of coping behaviours more holistically. Currently, the effects of parenting and parental are studied largely in rodents and within that almost inclusively in relation to understanding stress reactivity and parental separation. Imprinting is considered only in birds. Intergenerational transmission of early-life trauma across multiple generations is studied almost exclusively in humans, generally without regard for developmental processes beyond parental effects. Yet, each of the developmental processes reviewed here is likely to affect later-life coping behaviours, both independently and through complex interactions between different processes. Factors driving the development of behaviour cannot be well understood by studying one process in one group of animals, and another process exclusively in another group of animals with completely different life histories.

When considering vulnerability and reversibility of maladaptive coping behaviours, there is a need to distinguish between two different, though perhaps partially overlapping factors: sensitivity to receiving the necessary input (Bolhuis and Honey 1998; Zala et al. 2012) and sensitivity to negative influences such as stress or malnutrition (Krause et al. 2009; McClelland et al. 2011), although this distinction is rarely tested for empirically in studies on coping behaviours. Behaviours developed early in life can generally be applied later in life, but not all behaviours can be developed later in life. Often the pre-existing conditions simply do not exist or have developed (canalised) into a different direction. Alternatively, damage may have been done early in development that prohibits development in a certain direction. Deprivation of such necessary input during the sensitive stage may mean the juvenile will never develop the appropriate behaviour. Negative influences, on the other hand, can consist of (but not be limited to) momentary disruption of a developmental pathway and, as such, not necessarily be limited to a sensitive stage. For this reason, early life stress (ELS, Fuentes et al. 2014) should be considered within the context of the stressor, as evolutionary speaking, insufficient or abusive maternal care cannot be easily equated to lack of nutrition or lack of social interaction with conspecifics. Specifically, there is a need to move away from studying “stress” as a generic factor, and be more much focussed as to which developmental process is impacted by which early-life influence(s) specifically, and in what ecologically relevant way.

Although we have an increased understanding of the ultimate reasons why early-life environment affects developmental processes so strongly, and of some of the proximate mechanisms through which early-life development are shaped, there are still important gaps in our understanding, especially in the areas of threat perception and assessment, and non-genetic ways through which offspring copy parental coping behaviours. Further study in these areas, especially in the context of recent new thinking on evolutionary synthesis (Pigliucci 2009; Schoener 2011), will allow us to understand and perhaps even predict mismatches between environment and coping behaviours in animals, and ultimately, in ourselves.

## Electronic supplementary material

ESM 1 (DOCX 90 kb)
