# Supplementary material for: Why and how the early-life environment affects development of coping behaviours
Source: Behav Ecol Sociobiol. 2018 Feb 9;72(3):34. doi: 10.1007/s00265-018-2452-3 (PMC5805793; doi:10.1007/s00265-018-2452-3)
Supplement: Supplementary file 1 — (DOCX 90 kb) [file 265_2018_2452_MOESM1_ESM.docx]

## Title

Why and how the early-life environment affects development of coping behaviours

## Journal

Behavioral Ecology and Sociobiology

## Authors

M. Rohaa Langenhof (1) and Jan Komdeur (2)

## Affiliations

1) Behavioural Physiology and Ecology Group, Groningen Institute for Evolutionary Life Sciences, University of Groningen, Groningen, Netherlands; [m.b.w.langenhof@gmail.com](mailto:m.b.w.langenhof@gmail.com), ORCID: 0000 0003 3434 4357

2) Behavioural Physiology and Ecology Group, Groningen Institute for Evolutionary Life Sciences, University of Groningen, Groningen, Netherlands; [j.komdeur@rug.nl](mailto:j.komdeur@rug.nl),

# Supplementary material: Methodology and findings of literature survey

**Search strategy:** The ISI database and Google Scholar were searched late August 2014. One search was conducted to identify articles that addressed processes responsible for the development of coping (question 1), and the early-life influences on the development of coping behaviours such as boldness, shyness, avoidance, and predator-responses (question 2). A second search was done to identify studies on intergenerational transmission of coping behaviours (question 3). Manual searching was done on reference lists of included articles and relevant papers from this source were included as well. Searches were repeated in May 2017 and December 2017 to update our findings. 29 additional articles were found eligible. These articles were included in our review but not incorporated in the literature analysis below.

**Identification of eligible studies:** Studies in English were included when they presented studies that related any early-life influences to coping styles, correlations between behaviours and behavioural syndromes or personality. Results were excluded when they did not contain reference to behaviours aimed at mediating environmental challenges and included when they concerned behaviours that affect dealing with environmental challenge, which we feel includes social behaviour (Haller et al., 2014). Eligible studies on developmental processes (question 1) were included when they showed either empirical work demonstrating effects of such processes, or evolutionary theory indicating why such processes are expected to be important in development. Where multiple behaviours were reported as a result of a single environmental influence, such behaviours were grouped and included together rather than separately in Table 2. Studies were excluded if they did not report previously unreported empirical or theoretical work, or when they concerned behaviours not linked to coping with environmental challenges.

**Evaluation of eligible studies:** Data were entered into a spreadsheet and checked for discrepancies by an independent external researcher. Discrepancies were resolved by consensus. Important findings and implications from relevant studies were distilled and represented in sections 2, 3, 4 and 5.

**Data presentation and synthesis:** In studies included for questions 1 and 2, information was distilled on the kind of behaviours involved, and the type of environmental influence related to these behaviours. Empirical studies for questions 2 and 3 were displayed in a table, reviews were summarised. Where available, the developmental stage(s) during which environmental influences occurred were noted as well. In all cases, where provided, we recorded the following factors: study species, age of the measured individuals, rearing and experimental environment, as these factors have been shown to be of relevance in the expression of animal behaviour (see chapter 4). Such an overview allows us to categorise areas of interest in the literature, and gain insight in the breadth of relevant environmental factors. Linking this overview to the mechanisms through which environmental influences affect coping strategies, as we have done in figure 1, provides a better understanding of the likely processes underlying such effects and makes a step towards integrating ultimate and proximate lines of research.

**General results:** The literature search conducted by ourselves generated 761 unique results, 703 of which were excluded as not meeting the criteria. 498 titles did not concern coping behaviours as defined in our methodology, a further 93 did not concern early-life influences, and an additional 112 did not concern animals but humans or plants. From the literature, 89 titles/abstracts were included. An additional 31 were included after examining the references of included papers. Of all included papers, 62 were empirical studies and 27 were reviews. A total of 9 studies addressed question 1, 64 studies addressed question 2, and 16 studies addressed question 3. Several papers discussed both the effects of early-life conditions on individuals, and effects on their offspring. Rodents were the most heavily represented study species (27 studies, 19 of which were on rats), followed by various species of fish (13), birds (12), bigger mammals (9, including cows, horses, dogs) and monkeys (6). By far the majority of studies were conducted under laboratory conditions, with exception of a few who were raised in the wild (Kelley et al., 2005; Moretz et al., 2007; Roedel and Monclus, 2011; Sweeney et al., 2013; Wisenden et al., 2011).

| Table 1. What early-life influences affect the development of animal coping behaviours? Eligible empirical studies addressing effects of early-life influences on animal coping behaviours. | | | | | |  |
| --- | --- | --- | --- | --- | --- | --- |
| **Type** | **Early-life influence** | **Age** | **Coping behaviours** | **Species** | **Author, year** |  |
| 2 | parental licking and grooming | first 10 days | nurturing, bonding, emotional behaviours, social behaviours | prairie vole | Ahern 2009 |  |
| 3 | yolk reserves | larva at emergence | aggression, social dominance | rainbow trout | Andersson 2012 |  |
| 1 | habitat | 1-11 months | habitat choice | australian tiger snake | Aubret 2008 |  |
| 4 | peer-only rearing | infant | aggression, play and social behaviour, stress responsivity | rhesus macaques | Barr 2003 |  |
| 2 | maternal separation | pups (days 2-6) | response to novelty, emotional behaviours | rat | Biagini 1998 |  |
| 1 | housing environment | birth - suckling | responsivity, maze navigation, behavioural flexibility | pig | Bolhuis 2004 |  |
| 1 | housing environment | post weaning | aggression, play and social behaviour, activity | pig | Bolhuis 2005 |  |
| 1 | housing environment | suckling | chewing, manipulative and play behaviour | pig | Bolhuis 2006 |  |
| 5 | state and predator presence | larva stage | activity, boldness, foraging | damselfly | Brodin 2009 |  |
| 5 | darkness | first 6 days | shyness, predzator response | zebrafish | Budaev 2009 |  |
| 5 | immune challenge | mid and late development | activity, exploration, response to novelty | mallard ducks | Butler 2012 |  |
| 2 | maternal separation and handling | first 14 days | response to novely, exploration, foraging | rat | Caldji 2000 |  |
| 3, 4 | food availability and sibling competition | early rearing | exploration, aggression | great tit | Carere 2005 |  |
| 2 | mother's stress | gestation | anxiety, maternal behaviour | rat | Champagne 2006 |  |
| 3 | unpredictability in food supply | neonate fry | boldness, exploration | guppy | Chapman 2010 |  |
| 4 | communal rearing | postnatal | anxiety-like and maternal behaviour, dominance, aggression | mice | Curley 2009 |  |
| 5 | conspecific acoustic sexual signals | juveniles | aggression and dominance | field cricket | DiRienzo 2012 |  |
| 3, 4, 5 | conspecific presence, low food and perceived risk |  | exploration, boldness, aggression | killifish | Edenbrow 2013 | |
| 3 | unpredictable food supply | 40-90 days | boldness | seabass | Ferrari 2016 | |
| 4, 5 | gender, litter size, season of birth | first 10 days | confidence, aggression, physical and social engagement | german shepard dog | Foyer 2013 |  |
| 1, 2 | restricted bedding, substitute mother |  | anxiety, novelty seeking | long evens rat | Fuentes 2014 |  |
| 4 | litter sex ratio | 3 days postnatal | aggression, defensive burying, open field behaviour | rat | Gracceva 2011 |  |
| 4 | social experience |  | aggression, fear, sexual displays | black-headed gulls | Groothuis 1991 |  |
| 5 | tactile and/or visual stimulation | week 1 and 4 | passive avoidance reactions | rat | Gschanes 1998 |  |
| 5 | photoperiod |  | exploration, boldness, stress response | cavies | Guenther 2013 |  |
| 3 | litter size rank | day 1-3 | exploration, risk-prone behaviour | cavies | Guenther & Trillmich 2015 |  |
| 4 | social environment | lifetime | novelty, dispersal, behavioural plasticity | water striders | Han & Brooks 2014 |  |
| 2 | threat of infanticide |  | anxiety and exploration | mice | Heiming 2009 |  |
| 4 | relations with siblings | littering | behavioural style, stress response | rabbit | Hudson 2011 |  |
| 1 | habitat | rearing | courtship, aggression, boldness, foraging | spotted skiffia | Kelley 2005 |  |
| 2 | social instability for mothers | gestation | dominance, courtship, reactivity | guinea pig | Kemme 2008 |  |
| 3 | nutritional conditions | nestling / fledgeling | exploration, foraging, sensitivity to food restriction | zebra finch | Krause 2009 |  |
| 2, 4 | social and maternal isolation | infant | activity, abnormal behaviour | chimpanzee | Martin 2002 |  |
| 2, 4 | socialisation and father's behaviour | week 2 to 12 | response to familiarity and novel objects, boldness | cat | McCune 1995 |  |
| 2 | maternal separation | week 2 to 12 | stress behaviours | hose | Moons 2005 |  |
| 1 | habitat | from birth | shoaling, activity, boldness, foraging, aggressiveness | zebrafish | Moretz 2007 |  |
| 4 | social experience with dissimilar fish | juveniles | aggression, boldness, activity, stress behaviour | zebrafish | Moretz 2007 |  |
| 4 | social group size | nestling | stress response, exploratory behaviours | great tit | Naguib 2011 |  |
| 4 | population density | nymphal stage | boldness, aggression | field cricket | Niemela 2012 |  |
|  |  |  |  | zebra finch | Noguera 2015 |  |
| 2 | postnatal handling | first 21 days | reactivity to novelty and conflict | rat | Nunez 1996 |  |
| 3 | body size and growth |  | foraging behaviour across risk/boldness | pike | Nyqvist 2012 |  |
| 3 | nutrition | day 4 and 8 | exploration, stress handling | great tit | Van Oers 2015 |  |
| 5 | audiovisual playbacks | week 7 and 8 | exploration, fearfulness | dog | Pluijmakers 2010 |  |
| 1 | environmental enrichment | rearing | risk taking behaviour | salmon | Roberts 2011 |  |
| 4 | body mass and litter size |  | boldness, exploration, anxiety | rat | Roedel 2011 |  |
| 3 | body weight | early postnatal | anxiety, exploration | rabbit | Roedel 2011b |  |
| 2 | postnatal isolation | first 14 days | escape and avoidance | rat | Ruedi-Bettschen 2004 |  |
| 5 | predation |  | boldness, aggression | stickleback | Sih 2007 |  |
| 2 | mother isolation | days 2-10 | activity, orienting, risk taking | rat | Spivey 2008 |  |
| 1 | habitat |  | boldness, foraging aggression | spider | Sweeney 2013 |  |
| 4 | social isolation | day 23-38 (post weaning) | social recognition, anxiety | rat | Tanaka 2010 |  |
| 3 | environmental food conditions |  | boldness, activity, exploration | mustard leaf beetle | Tremmel 2013 |  |
| 4 | isolation | day 3-11 | coping with endotoxin, suckling behaviour | pig | Tuchscherer 2006 |  |
| 4 | isolation | week 4 and 5 | social behaviour, ambulation, rearing, self-grooming | rat | van der Berg 1999 |  |
| 2 | mother vs. artificial rearing | first 90 days | vigilance, escape, play and social behaviour | cow | Wagner 2013 |  |
| 5 | environmental bacteria | day 3 and 5 | anxiety-like behaviour | rat | Walker 2004 |  |
| 4 | habitat social complexity |  | cooperation and cognition | cleaner wrasse | Wismer 2014 |  |
| 1 | paternal personality |  | activity, boldness | zebra fish | Zajitschek 2017 |  |
| 5 | early cognitive stimulation | day 3 – day 35 | vigilance, escape attempts | red junglefowl | Zidar 2017 |  |
| Environmental influences are categorised as habitat (1), parenting (2), nutrition (3), social environment (4) or other influences (5). | | | | | |  |

| Table 2. How do coping behaviours of the parents transmit to their offspring through early-life influences? A short overview. | | | | |
| --- | --- | --- | --- | --- |
| **Author, year** | **Species** | **Parental influence** | **Age** | **Coping behaviours** |
| Babb 2014 | rat | chronic social stress (f0), impaired maternal care (f1) | lactation | maternal care (f1), social behaviour (f2) |
| Champagne and Meaney 2006 | rat | stress | gestation | maternal licking and grooming |
| Curley 2009 | mice | communal rearing | postnatal | anxiety-like and maternal behaviour, dominance, aggression |
| Fairbanks 1989 | vervet monkeys | mother-child experiences | first 6 months | mothering behaviours |
| Goerlich 2012 | domestic chicken | stress by social isolation | first 3 weeks | correct choices in learning test |
| Guesdon 2011 | quail | habitat hiding spaces |  | emotional and social reactivity of offspring |
| Maestripieri 2005 | rhesus monkey | maternal abuse | first 1 month | abusive parenting |
| McGhee 2012 | stickleback | maternal predator exposure | - | response to predator |
| Schuett 2013 | zebra finch | parental personality | - | exploratory type |

Analysis of all selected studies indicated several specific influences of interest, some of which have large areas of overlap. Parental care was the most prevalent early-life influence studied (18%), consisting of several different influences. Negative parental care was studied through maternal separation (6 studies) and impaired maternal care (4 studies). Other ways to ascertain the influence of parental care were through maternal licking and grooming behaviour, early social attachment to mothers, mother-child experiences, exposure to a substitute mother and parental personality traits. Parental care was especially studied in the context of intergenerational effects (63% of studies included for question 3), and much less so in the context of early-life effects alone (19% of studies included for question 2). Social influences were the main focus in 13 studies, through social experience / communal rearing, litter size and sex ratio, sibling competition and other relationships, and various expressions of social complexity and stability. Habitat conditions, measured mainly through wild vs. lab environments and the quality of housing conditions, were the focus in 10 studies. Less studied early-life influences were nutrition (7 studies), predation (5), abiotic conditions such as light (5), health (4) and sensory input (2). These influences were studied across different stages of early-life: in utero (10% of the studies that specified stage), first week post birth (27%), nursing to weaning (25%), nesting (16%), and fledging (2%). A number of studies took a broader approach of several intervals or a longer period up to six months post birth (10%).

Coping was measured across many different behaviours, most notably exploration (12% of all coping behaviours reported), aggression (10%), boldness (10%) and social behaviour (9%). Other commonly studied coping behaviours (more than 5 studies) included anxiety, stress responsivity, mothering behaviours, foraging, activity, social dominance, and response to novelty. Across these behaviours, there is some inconsistency in terminology, and some overlap between concepts. For example, stress responsiveness is an important coping behaviour, but was represented diversely by gestational stress (Champagne and Meaney, 2006), maternal separation (Biagini et al., 1998), social isolation (Tuchscherer et al., 2006), foraging demands (Kinnally et al., 2013), or not clearly defined at all in abstract or title. Certain behaviours were often found studied together. Overall, maternal behaviour, social deprivation and isolation are often considered in intergenerational studies; social stress, anxiety and aggression are mostly studied in rodents and related to human research; and exploration, boldness, and foraging are generally considered by biologists to understand animal personality.

**Processes for creating coping behaviours:** In answer to question 1, we found the following developmental processes mentioned within included reviews: maternal effects (Badyaev and Uller, 2009; Broadhurst, 1961; Fish et al., 2004; Reddon, 2012; Wisenden et al., 2011), imprinting (Hoffman and Ratner, 1973; Remy, 2010), habituation (Beach and Jaynes, 1954), conditioning (Groothuis and Mulekom, 1991; Hoffman and Ratner, 1973), perceptual learning (Beach and Jaynes, 1954) and social learning (Fairbanks, 1989; Maestripieri, 2005).

While genetics and epigenetics were mentioned often as proximate causes to the development of coping and as processes through which the environment affects behaviour, they represent a mechanistic, building-block level explanation rather than address the functional processes through which coping behaviours develop, and as such were not included as processes. Furthermore, the consensus among recent studies appears to be that environmental factors affect the expression of genetic material in a multitude of ways, and as such epigenetics likely underlie all developmental processes. Rather than exhaustively and mechanistically covering each process, an effort better left to experts within these fields, we summarised the most important processes in our paper.

**Early-life influences on development of coping behaviours:** In answer to question 2, search terms yielded 64 different mentions of early-life influences on coping behaviours, 52 of which from empirical studies (see table 1). Most prevalent in these studies were effects stemming in some way from the parents, either directly though parenting efforts, or indirectly through parent’s choice of rearing territory. From these 52 studies, we distilled four most important early-life influences: habitat conditions (studied in 9 studies), parents (14 studies), nutrition (7 studies), and social environment (18 studies), which we discuss in more detail hereafter. Other important early-life influences (11 studies) are abiotic factors such as photoperiod and season of birth, as well as direct influences to physical health such as predation, environmental bacteria and immune challenges.

**Processes for non-genomic transmission of coping behaviours**: In answer to question 3, nine empirical studies specifically showed parenting behaviours that affected the coping behaviour of subsequent generations, through maternal example and care, lack thereof, or abuse thereof. Within the included studies, all studies discussed effects relating to mothers, and only one related to both parents (Schuett et al., 2013).

Badyaev, A.V., and Uller, T. (2009). Parental effects in ecology and evolution: mechanisms, processes and implications. Philos. Trans. R. Soc. B Biol. Sci. *364*, 1169–1177.

Beach, F.A., and Jaynes, J. (1954). Effects of early experience upon the behavior of animals. Psychol. Bull. *51*, 239–263.

Biagini, G., Pich, E.M., Carani, C., Marrama, P., and Agnati, L.F. (1998). Postnatal maternal separation during the stress hyporesponsive period enhances the adrenocortical response to novelty in adult rats by affecting feedback regulation in the CA1 hippocampal field. Int. J. Dev. Neurosci. *16*, 187–197.

Broadhurst, P.L. (1961). Analysis of maternal effects in the inheritance of behaviour. Anim. Behav. *9*, 129–141.

Champagne, F.A., and Meaney, M.J. (2006). Stress During Gestation Alters Postpartum Maternal Care and the Development of the Offspring in a Rodent Model. Biol. Psychiatry *59*, 1227–1235.

Fairbanks, L.A. (1989). Early experience and cross-generational continuity of mother-infant contact in vervet monkeys. Dev. Psychobiol. *22*, 669–681.

Fish, E.W., Shahrokh, D., Bagot, R., Caldji, C., Bredy, T., Szyf, M., and Meaney, M.J. (2004). Epigenetic Programming of Stress Responses through Variations in Maternal Care. Ann. N. Y. Acad. Sci. *1036*, 167–180.

Groothuis, T., and Mulekom, L.V. (1991). The influence of social experience on the ontogenetic change in the relation between aggression, fear and display behaviour in black-headed gulls. Anim. Behav. *42*, 873–881.

Haller, J., Harold, G., Sandi, C., and Neumann, I.D. (2014). Effects of Adverse Early-Life Events on Aggression and Anti-Social Behaviours in Animals and Humans. J. Neuroendocrinol. *26*, 724–738.

Hoffman, H.S., and Ratner, A.M. (1973). A reinforcement model of imprinting: Implications for socialization in monkeys and men. Psychol. Rev. *80*, 527–544.

Kelley, J.L., Magurran, A.E., and Macı́as-Garcia, C. (2005). The influence of rearing experience on the behaviour of an endangered Mexican fish, Skiffia multipunctata. Biol. Conserv. *122*, 223–230.

Kinnally, E.L., Feinberg, C., Kim, D., Ferguson, K., Coplan, J.D., and Mann, J.J. (2013). Transgenerational Effects of Variable Foraging Demand Stress in Female Bonnet Macaques. Am. J. Primatol. *75*, 509–517.

Maestripieri, D. (2005). Early experience affects the intergenerational transmission of infant abuse in rhesus monkeys. Proc. Natl. Acad. Sci. U. S. A. *102*, 9726–9729.

Moretz, J.A., Martins, E.P., and Robison, B.D. (2007). Behavioral syndromes and the evolution of correlated behavior in zebrafish. Behav. Ecol. *18*, 556–562.

Reddon, A.R. (2012). Parental effects on animal personality. Behav. Ecol. *23*, 242–245.

Remy, J.-J. (2010). Stable inheritance of an acquired behavior in Caenorhabditis elegans. Curr. Biol. *20*, R877–R878.

Roedel, H.G., and Monclus, R. (2011). Long-term consequences of early development on personality traits: a study in European rabbits. Behav. Ecol. *22*, 1123–1130.

Schuett, W., Dall, S.R.X., Wilson, A.J., and Royle, N.J. (2013). Environmental transmission of a personality trait: foster parent exploration behaviour predicts offspring exploration behaviour in zebra finches. Biol. Lett. *9*, 20130120.

Sweeney, K., Gadd, R.D.H., Hess, Z.L., McDermott, D.R., MacDonald, L., Cotter, P., Armagost, F., Chen, J.Z., Berning, A.W., DiRienzo, N., et al. (2013). Assessing the Effects of Rearing Environment, Natural Selection, and Developmental Stage on the Emergence of a Behavioral Syndrome. Ethology *119*, 436–447.

Tuchscherer, M., Kanitz, E., Puppe, B., and Tuchscherer, A. (2006). Early social isolation alters behavioral and physiological responses to an endotoxin challenge in piglets. Horm. Behav. *50*, 753–761.

Wisenden, B., Sailer, C., Radenic, S., and Sutrisno, R. (2011). Maternal inheritance and exploratory-boldness behavioural syndrome in zebrafish. Behaviour *148*, 1443–1456.
